# Targeting the US21 viroporin of human cytomegalovirus by calcium channel blockers as a new antiviral strategy

**DOI:** 10.1016/j.crmicr.2026.100599

**Published:** 2026-04-19

**Authors:** Giulia Sibille, Davide Loggia, Gianluca Catucci, Alessandra Gilardino, Alessandra Fiorio Pla, Giovanna Di Nardo, Luca Munaron, Gianfranco Gilardi, Giorgio Gribaudo, Anna Luganini

**Affiliations:** Department of Life Sciences and Systems Biology, University of Turin, 10123 Turin, Italy

**Keywords:** Human cytomegalovirus, US21 viroporin, Calcium channel blockers, Drug repurposing, Molecular dynamics simulations, Antiviral activity, Cell migration, apoptosis

## Abstract

The Human cytomegalovirus (HCMV) US21 protein is a calcium-conducting viroporin that modulates intracellular Ca^2+^ homeostasis, safeguards cells from apoptosis, stimulates cell migration, and supports efficient HCMV replication. To validate pUS21 as a novel target for the identification of antiviral agents, *in silico* structure-based virtual screening was performed using its predicted structure to identify small molecules capable of engaging the inner part of the pore. Four dihydropyridine compounds (azelnidipine, efonidipine, lercanidipine, and niguldipine) were selected from 249 Calcium Channel Blockers (CCBs) in the DrugBank database. Molecular dynamics simulations of pUS21-ligand complexes predicted that the four selected CCBs formed dynamically stable and low-mobility interactions within the US21 pore, whereas the weak CCB binder felodipine remained highly mobile, supporting the predicted docking-based binding mode. The selected CCBs showed dose-dependent inhibition of HCMV replication in both fibroblasts and endothelial cells, with low micromolar EC_50_ values. Their antiviral effect was neither cell type- nor strain-dependent, as confirmed against two different clinical isolates, TRwt and VR1814, and was observed to be reduced against a US21-deficient virus, suggesting the specificity of pUS21 as a molecular target. Consistent with the predicted engagement within the pUS21 pore, CCBs prevented pUS21-mediated Ca^2+^ leakage from the endoplasmic reticulum and impaired both pUS21-induced cell migration and anti-apoptotic activity. Finally, drug combination studies revealed synergistic interactions between CCBs and maribavir treatment. Together, these findings support the hypothesis that clinically used CCBs may target pUS21 viroporin activity and hamper HCMV replication, thus offering a novel and promising antiviral strategy against HCMV, including drug-resistant strains.

## Highlights

• CCBs bind the inner pore of the HCMV US21 viroporin and abolish pUS21-mediated ER Ca^2+^ leakage.
• CCBs inhibit HCMV replication.
• CCBs prevent the cytobiological consequences of pUS21 expression.
• CCBs treatment restores ER Ca^2+^ levels to those of control cells.
• CCBs do not affect basal intracellular Ca^2+^ homeostasis.

## Graphical abstract

**Figure:**
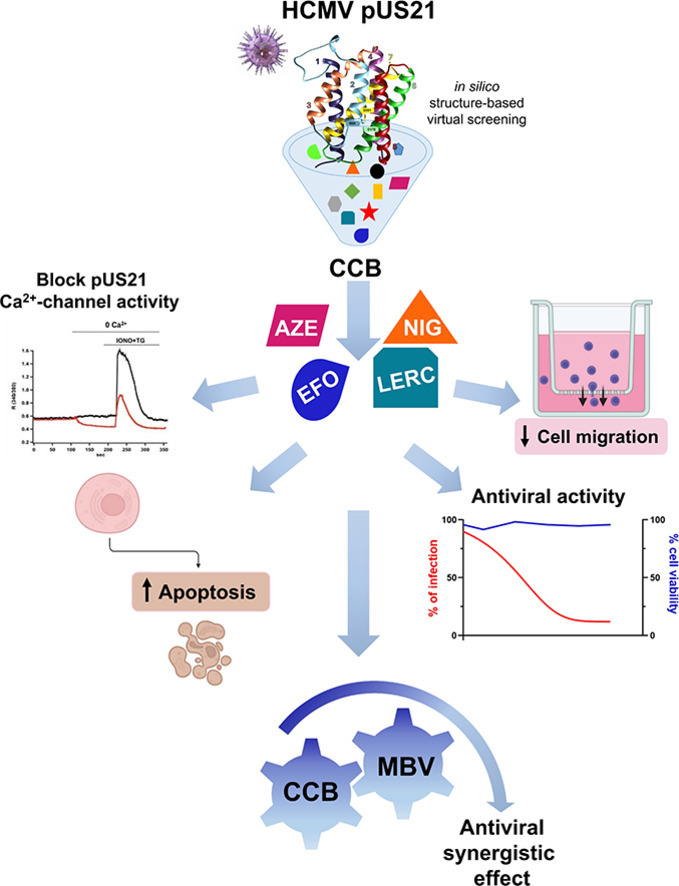

## 1 Introduction

Human cytomegalovirus (HCMV) is a ubiquitous β-herpesvirus that causes persistent infections and serious diseases in immunocompromised individuals. While primary infection is generally asymptomatic in healthy adults, congenital HCMV infection is the most common viral cause of sensorineural hearing loss and permanent neurological disability in newborns, with consequences that can emerge even years after birth (Griffiths et al., 2015; Britt, 2017). Moreover, in immunosuppressed patients, reactivation of latent HCMV is a critical event responsible for complications such as progressive retinitis, severe colitis, transplant rejection, and accelerated atherosclerosis (Mocarski et al., 2007; Griffiths, 2020). Furthermore, HCMV reactivation has been linked to the development of immunosenescence in the elderly and mortality in many groups of patients, including the general population (Griffiths, 2020). Despite its clinical relevance, current treatment options are limited to six antiviral agents: ganciclovir, valganciclovir, foscarnet, cidofovir, letermovir, and maribavir, which have various limitations, including hematological and renal toxicity, restricted use in high-risk clinical settings (excluding use in pregnancy and pediatrics), drug interactions, and, most importantly, the emergence of resistant strains, particularly in long-term transplant patients (Britt and Prichard, 2018; Piret and Boivin, 2019; Gourin et al., 2023).

Hence, in the absence of an approved vaccine, the identification of new virus-specific antiviral targets has become a priority. To address this need, regulatory viral proteins, such as viroporins, which are virus-encoded transmembrane proteins that form ion channels that dysregulate cellular ion homeostasis, thereby promoting viral replication, virion assembly, and cell-to-cell spread, may be considered as novel putative drug targets (Devantier et al., 2024; Alcaraz and Nieva, 2025; Qu et al., 2025). Indeed, viroporins from several viruses, such as M2 from Influenza, p7 from HCV, Vpu from HIV-1, DP1 and MgM from dengue virus and West Nile virus, respectively, and E from SARS-CoV-2, have already been recognized as potential drug targets (Nieva et al., 2012; Breitinger et al., 2022; Lahiri and Arkin, 2022; Devantier et al., 2024; Qu et al., 2025; Peng et al., 2025). Several studies have reported that clinically approved Calcium Channel Blockers (CCBs) can interfere with virus-induced Ca^2+^ signaling by targeting host cell Ca^2+^ channels, thereby impairing multiple steps of the infection cycle and exerting antiviral activity against relevant human viruses (Chen et al., 2019), such as the Influenza A virus, Ebola virus, and SARS-CoV-2 (Sakurai et al., 2015; Wang et al., 2022; Li et al., 2025). Overall, these findings highlight the importance of virus-encoded ion channels in viral replication and suggest that they may represent suitable novel drug targets for therapeutic intervention.

In this context, the characterization of the HCMV US21 protein (pUS21) as a Ca^2+^-permeable multitransmembrane viroporin localized in the endoplasmic reticulum (ER) is particularly relevant, as pUS21 promotes the release of Ca^2+^ from intracellular ER stores into the cytosol to set the stage for optimal viral replication (Luganini et al., 2018). Indeed, the pUS21-induced increase in cytosolic Ca^2+^levels has been observed to regulate pro-viral functions, such as anti-apoptotic activity that contributes to the survival of HCMV-infected cells, an increase in cellular ATP production to support viral synthesis, and the stimulation of infected cell migration, thereby facilitating the intra-host spread of the virus and its pathogenesis (Luganini et al., 2018, 2023).

In this study, to investigate the feasibility of the US21 viroporin as a putative viral target for the identification and development of anti-HCMV agents, we adopted an *in silico* structure-based virtual screening targeting the predicted pUS21 structure, which allowed us to identify four CCBs compounds endowed with potent inhibitory activity against HCMV replication, as well as on the biochemical and cytobiological activities of pUS21. These findings validate pUS21 as a novel HCMV-specific molecular target for antiviral drug discovery and pave the way for the development of highly specific drugs that are potentially safer and able to address the limitations of currently used drugs.

## 2 Materials and methods

### 2.1 Compounds

Azelnidipine (AZE), efonidipine (EFO), felodipine, lercanidipine (LERC), and staurosporine (STS) were from Merck. Maribavir (MBV) was from Selleck Chemical, and niguldinipide (NIG) from DBA. All compounds were resuspended in 100% DMSO at concentration of 50 mM. Tetracycline (TET) was purchased from Merck and resuspended in 100% ethanol at concentration of 1 mg/ml.

### 2.2 Structure-based virtual screening

The 3D model structure of pUS21 was obtained by Alphafold (Jumper et al., 2021). The model reliability was assessed using the transmembrane regions and the inner pore architecture by performing the plot of the Local Distance Difference Test (pIDDT). In addition, the Predicted Alignment Error (PAE) heatmap was assessed to evaluate the confidence in the relative positioning of residue pairs. The DrugBank database (https://go.drugbank.com/categories/DBCAT000574) was accessed to search for Calcium Channel Blockers (CCBs) using the code “voltage-dependent calcium channels”. A total of 249 CCBs were selected. The compound library was screened using AutoDock v4 (Morris et al., 2009), using a 3D model of pUS21 as the target, to estimate binding energies and dissociation constants for each CCB molecule. 3D images of the interactions were generated using UCSF Chimera 1.17.3, whereas 2D binding interactions between the molecules and the protein structures were further analysed by the Discovery Studio Client software (BIOVIA, Dassault Systèmes, Discovery studio client, 21.1, San Diego: Dassault Systèmes, 2021).

A previously established protocol was employed for the docking-based screening of drug candidates (Favretto et al., 2024). YASARA-integrated AutoDock (Morris et al., 1998) and AutoDock VINA (Trott and Olson, 2010) represent gold standard methodologies for predicting protein-ligand interactions. However, we selected VINA for our virtual screening campaign due to its superior computational efficiency and reduced computing time compared to the original AutoDock implementation. All docking calculations were performed using YASARA Structure (version 23.9.9) (Krieger and Vriend, 2014) running on Windows 11 Pro with an HP OMEN 880–102 nl workstation (Intel Core i7–8700 K, 2.8 GHz CPU, 16 GB RAM). A docking grid was defined encompassing extending 10 Å in all directions to establish the corresponding binding pocket. Docking simulations were executed with the YASARA-integrated AutoDock VINA algorithm employing the "dock_run.mcr" macro with 250 independent docking runs per ligand. This macro automatically performs ligand structure preparation, receptor-ligand docking, and ranking of predictions according to binding affinity. Results are automatically ranked by binding energy, where positive values indicate favorable binding interactions and negative values indicate unfavorable or non-binding conformations.

### 2.3 Molecular dynamics simulations

To investigate the dynamic stability of the pUS21 ligand complexes predicted by docking, molecular dynamics (MD) simulations were performed on the AlphaFold-derived model of pUS21 in its apo form (without ligand) and in complex with each dihydropyridine CCB (azelnidipine, efonidipine, lercanidipine, niguldipine, and felodipine, as a negative control). MD simulations were performed using YASARA Structure with the AMBER14 force field. A cubic simulation cell was automatically generated by the software around the protein, with a minimum distance of 10 Å from the protein in all directions (Gao et al., 2016). Each simulation was run for 150 ns at a physiological ion concentration (NaCl 0.9%) and a temperature of 310 K; water density was adjusted accordingly to the temperature to 0.99333 g/ml. A timestep of 2.5 fs was used, and trajectory snapshots were recorded every 250 ps. Simulation results were analyzed using the YASARA macro “md_analyze”. During trajectory analysis, we monitored the time evolution of the all atom root-mean-square deviation (RMSD) and the ligand RMSD with respect to the initial receptor-bound pose to assess the overall structural stability of US21 and of each complex. In addition, the ligand root-mean-square fluctuation (RMSF) was computed.

### 2.4 Cells and culture conditions

hTERT-immortalized HFFs fibroblasts (hT-HFFs), the human osteosarcoma U2OS cell line (ATCC HTB-96), and the stably transfected TET-inducible T-REx-U2OS-US21-HA cell line (Luganini et al., 2023) were grown as a monolayer in Dulbecco Modified Eagle’s Medium (DMEM) (Euroclone), supplemented with 10% tetracycline-reduced Fetal Bovine Serum (Euroclone). T-REx-U2OS-US21-HA cell lines were grown in the presence of 2 μg/ml blasticidin and 500 μg/ml zeocin (Life Technologies, Carlsbad, *CA*, USA). To induce expression of pUS21-HA proteins, tetracycline (1 μg/ml) was added for 48 h. Human dermal microvascular endothelial cells (HMVECs) (CC-2543) were obtained from Clonetics and cultured in endothelial growth medium (EGM) as described previously (Bronzini et al., 2012; Luganini et al., 2017).

### 2.5 Viruses

The wild-type ganciclovir (GCV)-resistant TR (TRwt) virus (Smith et al., 1998) and its derivatives, TRΔUS21 and TRUS21stop, were reconstituted by transfecting hT-HFFs with the corresponding BAC as previously described (Luganini et al., 2018). The reconstituted wild type TR-BAC generated infectious virus that retained the ability to infect endothelial and epithelial cells, as well as monocytes and macrophages (Murphy et al. 2003; Ryckman et al., 2006; Bronzini et al., 2012; Luganini et al., 2017, 2018).

GCV-sensitive HCMV VR1814 is a clinical isolate recovered from a cervical swab of a pregnant woman (Revello et al., 2001).

HCMV strains were propagated on human fibroblasts and titrated by the indirect immunoperoxidase staining procedure on hT-HFF using a mouse monoclonal antibody (MAb) anti-HCMV IEA (P1215, Virusys Corporation) (Bronzini et al., 2012; Cavaletto et al., 2015; Luganini et al., 2017).

### 2.6 Cell cytotoxicity assay

The effect of test compounds on cell viability was determined in hT-HFFs, HMVECs and T-REx-U2OS-US21-HA cells seeded in p96-well (12,000 cells/well). After 24 h, cells were treated with compounds at concentrations starting from 300 μM and then serially diluted in culture medium, or with DMSO alone starting from 0.6% v/v as the vehicle control. For cell viability of drug combination studies, the control with DMSO was from 0.07%. Then, after two days of treatment for T-REx-U2OS-US21-HA cells or five days for hT-HFFs and HMVECs, cell cultures were processed for the measurement of cell viability using the 3-(4,5-dimethylthiazol-2-yl)-2,5-diphenyltetrazolium bromide (MTT) method (Luganini et al., 2008). GraphPad Prism software version 8.0 was used to determine the compound concentration that produces 50% of cytotoxicity concentration (CC_50_).

### 2.7 Antiviral assay

The antiviral activity of CCBs was evaluated against VR1814 and TRwt by means of the focus-forming reduction assay (FFRA) procedure. Briefly, hT-HFFs were seeded on 96-well plates (20,000 cells/well) and, after 24 h, treated with different concentrations of compounds (maximum concentration 20 μM, or 100 μM for felodipine) 1 h prior to and during viral infection (50 PFU/well). Control HCMV-infected cells were exposed to vehicle DMSO at 0.04%, or 0.2% for felodipine). After virus adsorption (2 h at 37 °C), virus inoculum was removed and infected cell cultures were incubated in medium containing the corresponding compounds plus 1.2% (w/v) methylcellulose (Merck) and 5% (v/v) FBS. At 96 h post infection (p.i.), cell monolayers were fixed and subjected to indirect immunoperoxidase staining (IPA) with the HCMV IEA mAb (diluted 1:150). Viral foci were microscopically counted, and the mean plaque counts for each drug concentration were expressed as a percentage of the mean plaque counts of control virus (DMSO). GraphPad Prism software was used to determine the concentration of compounds producing 50 and 90% reductions in viral titers (EC_50_ and EC_90_).

For time-of-addition experiments, hT-HFFs cells were seeded at a density of 20,000 cells/well in 96-well plates. The following day, cells were treated with CCBs at a concentration of their 2 x EC_50s_ from −1 h to 0 h prior to HCMV infection (MOI of 0.1 PFU/cell) (pre-treatment, PRE-T); or during infection (adsorption stage, from 0 h to 3 h, co-treatment, CO-T); or after virus adsorption (from 3 h to 6 days (d) p.i., post-treatment, POST-T); or throughout the experiment from −1 h prior to HCMV to 6 d p.i. (full treatment, Full-T). DMSO was used as control at 0.01%. At 6 d p.i., the cell supernatants were harvested and titrated for HCMV infectivity as described above.

### 2.8 Drug combination studies

To evaluate the combined effects of each CCBs and maribavir (MBV) on HCMV TRwt replication, FFRAs were performed as described above using 0.25-, 0.5-, 1-, 2-, 4-fold their respective EC_50_ for each combination of AZE, EFO, LERC, NIG and MBV at equipotent ratio. To evaluate the antiviral activity of each CCB alone, the control HCMV-infected cells were exposed to DMSO at the highest concentration used of each CCB used, resulting in 0.02% for AZE, 0.03% for EFO and LERC, 0.01% for NIG and 0.04% for MBV). For 2-drug combination, the control HCMV-infected cells were exposed to DMSO starting at concentrations of 0.05% for AZE + MBV and NIG + MBV, 0.07% for EFO + MBV, and 0.06% for LERC + MBV. The 2-drug combination effects were assessed using the Chou-Talalay method (Chou, 2006, 2010) according to mass-action law based dynamic theory computed in the CalcuSyn software version 2.0 (Biosoft, Cambridge, UK) (Chou and Martin, 2005).

### 2.9 Analysis of pUS21 expression

For immunoblotting, total cell proteins were extracted either at different timepoints following tetracycline stimulation for T-REx-U2OS-US21-HA cells or at 48 h post-transfection for U2OS cells and analyzed by immunoblotting as previously described (Luganini et al., 2017, 2018, 2023). pUS21 proteins were immunostained using a rat anti-HA mAb conjugated to horseradish peroxidase (clone 3F10, Roche) diluted to 1:100.

Immunodetection of tubulin with a mouse mAb (clone TUB 2.1, Sigma) was used as a control for cellular protein loading.

### 2.10 Cytosolic Ca^2+^ quantification

Calcium measurement was determined applying the Ca^2+^ add back protocol as previously described (Luganini et al., 2023). Briefly, non-induced (NI) or induced (I) T-REx-U2OS-US21-HA cell lines for 48 h with tetracycline, plated at a density of 5000 cells/cm^2^ on glass coverslips, were loaded (45 min at 37 °C) with 2 μM Fura-2 AM (Invitrogen, Waltham, MA, USA) and excited at two alternating wavelengths: 340 and 380 nm. Fluorescence emission was captured using a Nikon Eclipse TE-2000S inverted microscope equipped with the MetaFluor Imaging System (Molecular Devices, San Jose, *CA*, USA). Ratiometric cytosolic Ca^2+^ ([Ca^2+^]_c_) quantification was expressed as the ratio (R) of fluorescence emitted at 510 nm for the two excitation wavelengths. Images were acquired every 3 s. Ca^2+^ imaging analysis was conducted by peak amplitude quantification using Clampfit 11.1 (Axon pClamp, Molecular Devices). Responses with a ΔR340/380 > 0.05 were taken in account. To assess the ER Ca^2+^ content in T-REx-U2OS-US21-HA NI or I cells, depletion of the ER Ca^2+^ pool was triggered using IP3mix in the absence of extracellular Ca^2+^ (0 Ca^2+^). IP3mix consisted of thapsigargin (2 μM), histamine (100 μM), and ATP (100 μM). The experiments were performed in acute treatment in presence of CCBs or DMSO, as control at 0.01%.

### 2.11 Migration assays

For migration assay, T-REx-U2OS-US21-HA cells were left non-induced (NI) or induced (I) with TET (1 μg/ml) for 48 h. Then, cells were trypsinized, seeded to the upper side of 8 μm PET 24-well multiwell inserts system for 7 h (37 °C in 5% CO_2_) and maintained in DMEM (200 μl) plus the indicated CCBs (with or without TET). The lower chamber was filled with DMEM supplemented with 10% FBS, with or without TET, plus CCBs at concentration of their 2 x EC_50s_ (800 μl). DMSO was used as control at 0.01%. Each experimental condition was performed in triplicate. Cells remaining on the upper surface of the inserts were removed using a cotton swab. To quantify the cells that migrated to the bottom of the inserts, the procedure described by Luganini et al., 2023 was carried out. The number of cells was counted for at least ten different fields by an Olympus IX50 fluorescence microscope equipped with Image-Pro Plus software. Chemotactic migration assays of hT-HFFs was performed by maintaining the cells in DMEM supplemented with 0.5% FBS (starvation medium) at 24 h prior to infection. Then, cells were mock-infected or infected with TRwt, or TRΔUS21 at an MOI of 1. At 30 h p.i., infected cells were treated with CCBs in starvation medium for 24 h before trypsinisation and seeding into the upper part of 8 μm pore PET multiwell inserts. The lower chamber was filled with DMEM supplemented with 10% FBS, and CCBs, where indicated. Cells were then allowed to migrate through the PET membrane for 24 h at 37 °C in an atmosphere containing 5% CO_2_.

### 2.12 Apoptosis caspase-3/7 assay

For transient expression of pUS21-HA in U2OS cells, cell cultures (18,000 cells/well) were transfected with 350 ng of pcDNA3.1 (Invitrogen) and either pcDNA3.1-US21HA (pUS21HAwt), pcDNA3.1-US21HA-D178N (pUS21HA-D178N), or pcDNA3.1-US21HA-D201N (pUS21HA-D201N) (Luganini et al., 2018) using *Lipofectamine*™ 3000 Reagent (ThermoFisher) following *manufacturer*'s instructions. At 6 h post-transfection, cells were treated with AZE, EFO, LERC, and NIG in medium containing 10% FBS for 48 h. Control cells were exposed to DMSO at 0.01%. Then, cells were washed three times in DMEM, and, where indicated, staurosporine 3 μM and/or CCBs were added in DMEM + 2% FBS, for 4 h at RT before measuring caspase-3 and caspase-7 activity. Levels of caspase-3/7 were measured using the Caspase-Glo 3/7 substrate (Promega). Luminescence was quantified using the Glomax Explorer multimode plate reader (Promega).

### 2.13 Statistical analysis

All data were generated from at least three independent experiments performed in triplicate. Statistical analysis was performed using GraphPad Prism software version 8.0 (San Diego, *CA*). The results from the antiviral assays are shown as the means ± SD. Data from cytosolic Ca^2+^ quantification experiments were analyzed by an unpaired *t*-test; data from migration assays were analyzed by a Kruskal-Wallis multiple comparison test; data from caspase-3/7 assay were analyzed by a Dunnett’s multiple comparison test. Data were considered to be statistically significant for p values ≤ 0.05. Significance was set at: *, p < 0.05; **, p < 0.005; ***, p < 0.001; ****, p < 0.0001.

## 3 Results

### 3.1 In silico structure-based virtual screening identifies Calcium Channel Blockers targeting pUS21

**Fig. 1:**
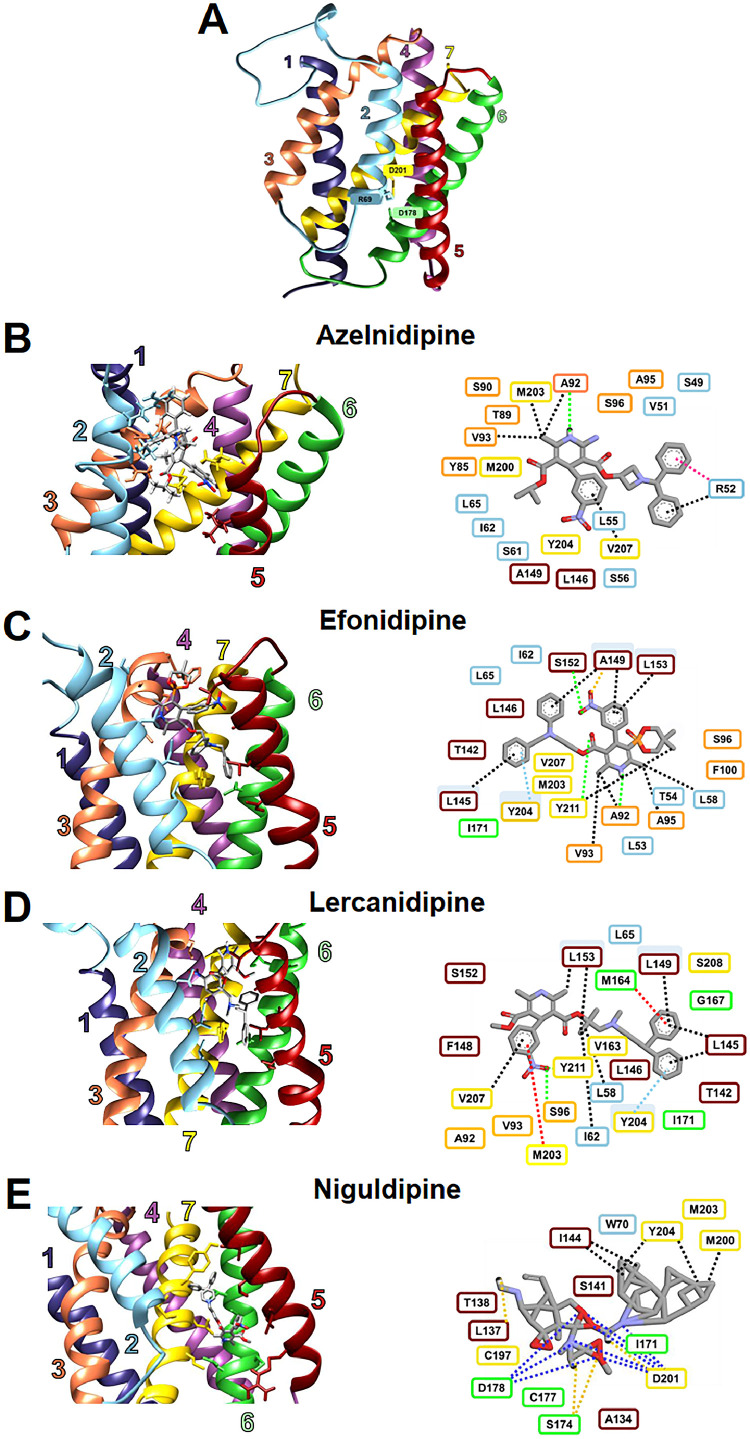
**Representation of the predicted interactions between the selected CCBs and pUS21. (A)** Structural model of pUS21 in the open conformation, as predicted by AlphaFold. **(B-E)** Simulated interactions of azelnidipine **(B)**, efonidipine **(C)**, lercanidipine **(D)**, and niguldipine **(E)** within the pUS21 internal pore. The 3D model of 7TMD pUS21 was generated using AlphaFold software, and molecular docking analyses were performed using AutoDock v4 to predict the binding interactions between pUS21 and selected CCBs. 3D images of the interactions were generated using UCSF Chimera 1.17.3 (left), whereas 2D interaction diagrams were produced using Discovery Studio software (right). The contour color of each amino acid residue matches that of the corresponding TMD of the pUS21 structural model (**A**), while each dashed line that represents a ligand-residue interaction is color-coded by the interaction type as follows: green for conventional hydrogen-bonds, pink for π-cation bonds, yellow for carbon-hydrogen bonds, red for π-sulfur, light blue for π-π stacking, black for π-alkyl/alkyl, and blue for attractive charge. Amino acid residues shown without dashed lines are involved in van der Waals interactions with the ligand.

The predicted open conformation of the 7TMDs pUS21 was generated AlphaFold using (Fig. 1A). Before starting with the virtual *in silico* screening, to validate Alphafold model, we analysed the Local Distance Difference Test (pLDDT) profile and the Predicted Alignment Error (PAE) heatmap. The pLDDT profile of the AlphaFold model shows values > 60 (on a scale ranging from 0 to 100) for the residues lining the predicted pore region (Fig. S1), indicating a consistent and structurally coherent definition of the transmembrane core. The PAE heatmap is shown in Fig. S2 and indicates that most of the residue pairs display relatively low expected position errors (in the range of 5–10 Å), supporting a reliable global arrangement of the transmembrane helices. Since the validation was successful, the pUS21 3D-model structure was used to screen a library of 249 Calcium Channel Blockers (CCBs) using the AutoDock software on a library of 249 Calcium Channel Blockers (CCBs). In particular, the internal region of the predicted pUS21 pore was exploited as the protein target site for small molecule docking. This *in silico* screening selected six different CCBs molecules as suitable binding partners of pUS21, based on the best binding energy values (Table 1). Among these CCBs, the antihypertensive dihydropyridines azelnidipine (AZE), efonidipine (EFO), lercanidipine (LERC), and niguldipine (NIG) were chosen for further analyses considering their binding energy, dissociation constant and commercial availability (Table 1). In addition, felodipine was included as a negative reference compound, as it has a relatively weaker binding affinity than other dihydropyridines.

**Table 1:** Results of the *in silico* structure-based virtual screening of a Calcium Channel Blockers (CCBs) library using the predicted structures of pUS21.

Fig. 1 (B-E) shows the details of the predicted interactions of the four selected CCBs within the pUS21 pore, while the corresponding interaction types and involved residues, as annotated by Discovery Studio (DS), are summarized in Table 2. In detail, azelnidipine was found bind to the pUS21 channel pore primarily through hydrophobic complementarity driven, driven by extensive van der Waals and π-alkyl/alkyl interactions provided by the TM6 and TM7 domains. In addition, an electrostatic interaction with the TM2 residue R52 (charge-π/alkyl) may contribute to stabilize the ligand within the pore. Overall, the AZE-pUS21 interactions define a hydrophobically driven binding mode that is influenced by the local conformational arrangement of the binding site. Efonidipine displayed a mixed interaction profile characterized by specific anchoring through three conventional hydrogen bonds with residues A92 (TM2), S152 (TM6), and Y211 (TM7), combined with extensive hydrophobic stabilization mediated by van der Waals contacts. Moreover, a π-π stacking interaction with residue Y204 on TM7 supported a defined orientation of the aromatic moiety within the channel pore. This interaction pattern indicated a well-organized and specific EFO binding mode.

**Table 2:** Aminoacids residues of pUS21 predicted to be involved in distinct chemical interactions with CCBs.

	Van der Waals	Conventional hydrogen-bond	Carbon-hydrogen-bond	π-sulfur		π-π stacking	π-alkyl/ alkyl	Attractive charge
**Azelnidipine**	S49, V51, L55, S56, S61, I62, L65, Y85, T89, S90, A95, S96, L146, A149, M200, Y204	A92					R52, V93, M203, V207	R52
**Efonidipine**	L53, T54, 162, L65, S96, F100, T142, L146, I171, M203, V207	A92, S152, Y211	A149			Y204	L58, A92, V93, A95, L145, A149, L153, Y211	
**Lercanidipine**	L65, A92, T142, L146, F148, S152, V163, G167, I171, S208	S96, Y211	V93		M164, M203	Y204	L58, I62, L145, A149, L153, V207	
**Niguldipine**	W70, R134, T138, S141, I171, C177, C197, M203		L137, S174, D201				I144, M200, Y204	D178, D201

Among the four selected CCBs, lercanidipine showed the most complex and diversified interaction network, which included multiple specific bonds such as multiple hydrogen bonds, C—H bonds, π-sulfur interactions, π-π stacking, and van der Waals interactions. Notably, π-sulfur interactions with residues M164 (TM6) and M203 (TM7), together with π-π stacking in Y204 (TM7), likely contribute to the stable accommodation of the aromatic rings of LERC within the pore. Finally, the binding of niguldipine is mainly characterized by two electrostatic interactions with D178 (TM6) and D201 (TM7), two aspartic acid residues that have been reported to be required for the Ca2+ channel activity of pUS21 (Luganini et al., 2018); thus, NIG binds in a pUS21-specific and preferential manner.

### 3.2 Molecular dynamics simulations of selected CCBs

**Fig. 2:**
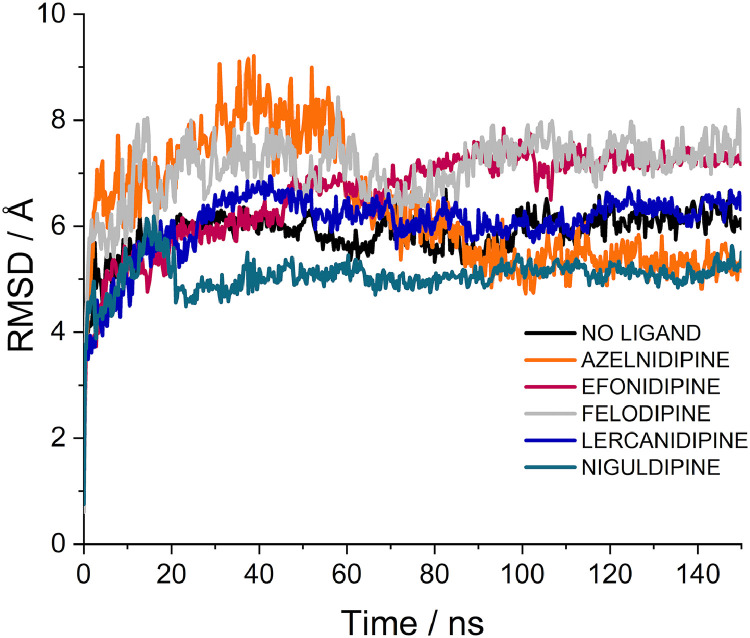
**Molecular dynamics simulation of pUS21 in the absence (no ligand) and in the presence of CCB.** All-atom RMSD of pUS21 over time (ns) in the apo state (no ligand) and in complex with the best CCB binders, azelnidipine, efonidipine, lercanidipine, or niguldipine is shown. Felodipine was included as a weak CCB binder.

**Fig. 3:**
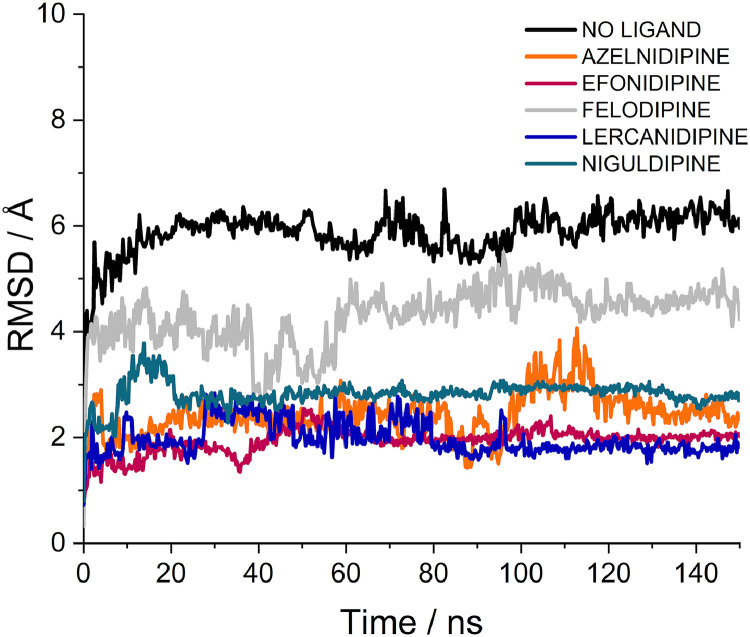
**Molecular dynamics simulation of pUS21 in complex with selected CCBs.** RMSD over time is shown for azelnidipine, efonidipine, lercanidipine, and niguldipine. Felodipine was included as a weak CCB binder. The data were obtained by performing a superposition of the protein structure.

Previous studies have clearly indicated how molecular dynamics (MD) simulations can be used to complement and validate docking experiments (Weng et al., 2022; Wang et al., 2025). To investigate the structural dynamics of the four CCBs selected by structure-based virtual screening, MD simulations were conducted for 150 ns on each of the CCB-pUS21 complexes, including the weak CCB binder, felodipine (Table 1), as a negative control. For each complex, we monitored the time evolution of all-atom protein root-mean-square deviation (RMSD) (Fig. 2) and ligand RMSD (Fig. 3) to evaluate both the global stability of the protein and the behaviour of each ligand in the pore. The results indicated that pUS21, in the presence and absence of CCBs, maintained a stable RMSD with average values between 5.8 Å and 7.1 Å. The relatively high RMSD values observed for pUS21 likely reflect intrinsic system properties, such as the fact that pUS21 is a multi-transmembrane protein modeled by AlphaFold and may undergo structural relaxation during MD, particularly in the flexible regions. Nevertheless, the RMSD values plateaued over time, indicating equilibration rather than instability. Overall, these values are consistent with global flexibility and are not uncommon for predicted membrane protein models in simplified-simulation environments.

The ligand RMSD traces revealed a clear distinction between the selected CCBs and the negative control felodipine. Efonidipine, lercanidipine, and niguldipine rapidly reached a plateau after an initial equilibration phase and then fluctuated within a narrow RMSD range (Fig. 3), indicating that these ligands remained tightly anchored in a well-defined pose inside the pUS21 pore over the simulated timescale. Azelnidipine showed somewhat larger but still relatively contained RMSD fluctuations (Fig. 3), compatible with a stable, though slightly more flexible, binding mode. In contrast, felodipine exhibited higher and more variable ligand RMSD profiles (Fig. 3), which indicated increased conformational drift and reorientation out of the channel. This behaviour was therefore consistent with its classification as a weak binder in docking analyses.

To further quantify ligand mobility, the average ligand RMSF values were calculated over the production phase of the simulations. Efonidipine, lercanidipine, and niguldipine showed the lowest average RMSF values (2.36 Å, 2.25 Å, and 2.15 Å, respectively), in agreement with their low and stable RMSD traces and indicative of a constrained motion around a specific binding mode within the pore. Azelnidipine and felodipine exhibited intermediate RMSF values (5.78 Å and 5.73 Å, respectively). Notably, despite similar RMSF magnitudes, their dynamic behaviour differed, as reflected by their RMSD profiles (Fig. 3) and binding persistence within the pore. In fact, despite its relatively high RMSF, azelnidipine maintained a stable positioning within the pUS21 pore over time, without the pronounced conformational drift observed for felodipine. Overall, these MD data support the mechanistic plausibility of the docking poses by showing that the four best CCBs form dynamically stable complexes with pUS21, whereas the negative control, felodipine, remains highly mobile and does not maintain persistent engagement of the viroporin inner pore.

### 3.3 The selected CCBs inhibit HCMV replication

**Fig. 4:**
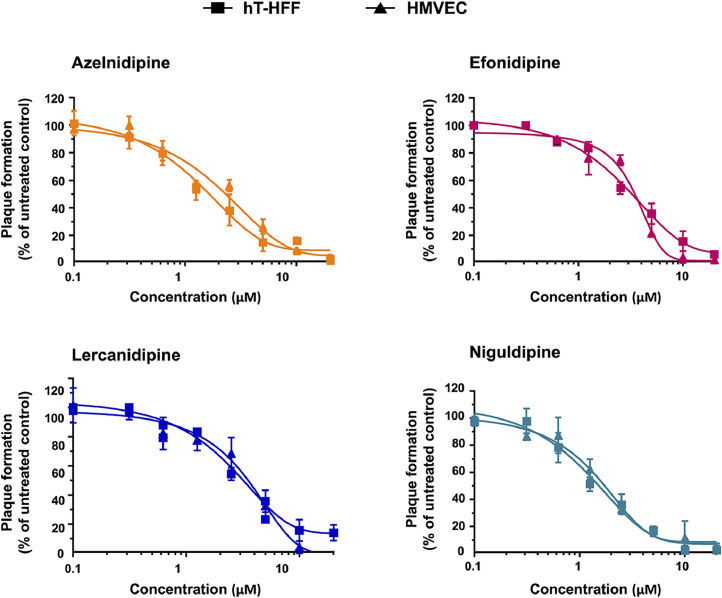
**CCBs selected by binding to pUS21 inhibit HCMV replication.** Focus forming reduction assays (FFRAs) were performed in hT-HFFs and HMVEC cells infected with TRwt (50 pfu/well) and treated with different concentrations of CCBs 1 h before, during, and after infection. At 96 h p.i., the viral foci were microscopically counted and the mean plaque counts for each drug concentration were expressed as a percent of the mean count of the control cultures treated with the DMSO vehicle. Data are shown as the means ± SD of three biological experiments, each performed in two technical replicates.

**Fig. 5:**
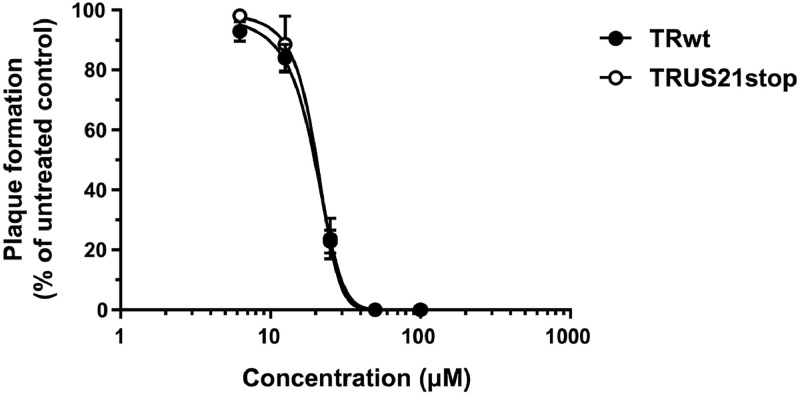
**The weak pUS21 binder felodipine barely inhibit HCMV replication.** hT-HFFs were infected with HCMV TRwt and TRUS21stop (50 pfu/well) and treated with increasing concentrations of felodipine 1 h before, during, and after infection to perform Focus forming reduction assays (FFRAs). At 96 h p.i., the viral foci were microscopically counted and the mean plaque counts were expressed as a percent of the mean count of the control cultures treated with the DMSO vehicle. Data are shown as the means ± SD of three biological experiments, each performed in two technical replicates.

To investigate the antiviral activity of the CCBs selected based on their predicted ability to bind within the pUS21 pore, focus-forming reduction assays (FFRAs) were performed using the GCV-resistant HCMV TRwt strain (Smith et al., 1998) in hTERT-immortalized fibroblasts (hT-HFFs) and human dermal microvascular endothelial cells (HMVECs). As depicted in Fig. 4, all four tested CCBs showed a dose-dependent inhibitory effect on TRwt replication in both fibroblasts and endothelial cells. The measured EC_50s_ values were in the low-micromolar range, regardless of the cell type used as the host cell system (Table 3). Moreover, the anti-HCMV activity of the CCBs was not due to cytotoxicity, as their cytotoxic concentrations 50 (CC_50_) values in both hT-HFFs and HMVECs were higher than 200 μM (Table 3), demonstrating that their antiviral effect was not due to non-specific cytotoxic effects. This led to favorable Selectivity Index (SI) values for all four tested compounds (Table 3). Notably, the TRUS21stop virus, in which the US21 ORF was inactivated by the insertion of a stop codon near the N terminus (Luganini et al., 2018), showed EC_50_ values in hT-HFFs for all the tested CCBs increased by more than two-fold compared to those of TRwt (Table 3). This observation suggested that the anti-HCMV activity of the four CCB molecules may be due, at least in part, to their predicted ability to bind to pUS21, thus interfering with its function and, consequently, hindering HCMV replication. To further support this hypothesis, we examined the effect of the weak CCB binder, felodipine (Table 1), on HCMV TRwt replication. As shown in Fig. 5, felodipine exerted a poor antiviral activity which resulted in minimal reduction of viral replication with EC_50_ values of 19.45 μM for TRwt and 19.36 μM for TRUS21stop. This finding clearly indicated that the anti-HCMV activity of AZE, EFO, LERC, and NIG is not a general feature of dihydropyridine CCBs but is consistent with an interaction with the pUS21 pore, as predicted by structure-based screening.

**Table 3:** Antiviral activity of selected CCBs against HCMV TRwt and TRUS21stop.

CCB	Cell line	Virus	EC_50_ (μM)^a^	EC_90_ (μM)^b^	CC_50_ (μM)^c^	SI^d^
**Azelnidipine**	hT-HFF	TRwt	1.53 ± 0.32	12.10 ± 0.27	192.02 ± 20.46	125.50
	HMVEC		2.12 ± 0.14	9.53 ± 0.76	341.05 ± 20.08	160.87
	hT-HFF	TRUS21stop	5.46 ± 0.17	19.36 ± 0.06	192.02 ± 20.46	35.16
**Efonidipine**	hT-HFF	TRwt	3.18 ± 0.07	14.21 ± 1.67	339.71 ± 20.10	106.82
	HMVEC		3.55 ± 0.13	6.58 ± 0.91	295.41 ± 11.01	83.21
	hT-HFF	TRUS21stop	7.11 ± 0.05	> 20	339.71 ± 20.10	47.77
**Lercanidipine**	hT-HFF	TRwt	3.08 ± 0.28	12.65 ± 1.60	126.54 ± 18.76	41.08
	HMVEC		3.32 ± 0.38	5.90 ± 0.11	285.83 ± 19.06	86.09
	hT-HFF	TRUS21stop	7.60 ± 0.11	19.92 ± 0.50	126.54 ± 18.76	16.65
**Niguldipine**	hT-HFF	TRwt	1.26 ± 0.31	7.33 ± 2.54	241.16 ± 23.89	191.39
	HMVEC		1.15 ± 0.29	7.24 ± 0.59	205.16 ± 23.30	178.40
	hT-HFF	TRUS21stop	3.51 ± 0.22	13.00 ± 0.63	241.16 ± 23.89	68.70

a EC_50_, compound concentration that inhibits 50% of TRwt strain replication, as measured by FFRA in hT-HFFs or HMVECs by FFRAs, and TRUS21stop replication in hT-HFFs.

b EC_90_, compound concentration that inhibits 90% of TRwt strain replication, as determined by FFRA in hT-HFFs or HMVECs by FFRAs, and TRUS21stop replication in hT-HFFs.

c CC_50_, compound concentration that produces 50% of cytotoxicity, as determined by cell viability assays in hT-HFFs and HMVEC cells. Reported values represent the means ± SD of data derived from three experiments in duplicate.

d SI, selectivity index determined as the ratio between CC_50_ and EC_50_.

**Fig. 6:**
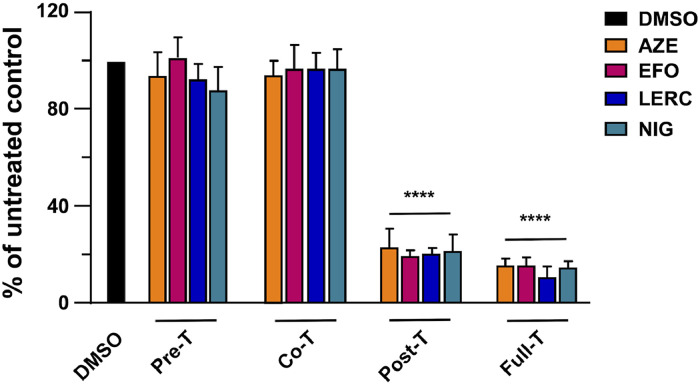
**CCBs target HCMV replication at a post-entry stage of the replication cycle.** hT-HFFs cells were infected with TRwt at an MOI of 0.1 for 6 d p.i. and where indicated, treated with 2 x EC_50_ of AZE, EFO, LERC, and NIG prior to infection (from −1 to 0 h prior, PRE-T), or during the infection (from 0 to 3 h p.i., CO-T); or after virus infection (from 3 to 6 d p.i., POST-T); or from −1 h prior infection to 6 d p.i. (FULL-T). Control HCMV TRwt-infected cells were exposed to vehicle DMSO only. At 6 d p.i., infectious virus released into culture supernatants was quantified by plaque assay on hT-HFFs cells. The data shown are the mean ± SD of two biological independent experiments, each performed in three technical replicates and analyzed by the Dunnett’s multiple comparison test. **** p < 0.0001 vs calibrator sample (DMSO-treated TRwt-infected cells).

Then, to rule out the possibility that the anti-HCMV activity of CCBs was strain-specific, FFRAs were performed on hT-HFFs and HMVECs infected with the endotheliotropic VR1814 strain. As shown in Table 4, the four CCBs hindered VR1814 replication with EC_50_ values similar to those measured with TRwt, suggesting that the antiviral activity of the selected CCBs against HCMV was independent of the virus strain or the cell type. Next, time-of-drug-addition (ToA) experiments were performed to identify the stage of the HCMV replication cycle targeted by the four CCBs. hT-HFF cells were treated with the CCBs prior to infection (PRE-T), during virus adsorption (CO-T), after viral entry (POST-T), or throughout the entire experiment (FULL-T). At 6 d p.i., viral replication was then quantified and compared with DMSO-infected cells. As shown in Fig. 6, none of the compounds displayed antiviral activity when added during adsorption (PRE-T) or entry (CO-T), indicating their inability to interfere with HCMV attachment or entry into target cells. In contrast, all CCBs significantly reduced HCMV infection by approximately five-fold when added at a post-entry stage (POST-T) or left on cells for the entire experiment. This pattern is consistent with the inhibition of a late step in the viral replication cycle and further supports the hypothesis that CCBs may exert their antiviral activity by interfering with the functions of the late US21 protein.

**Table 4:** Antiviral activity of selected CCBs against HCMV VR1814.

CCB	Cell line	Virus	EC_50_ (μM)^a^	EC_90_ (μM)^b^	CC_50_ (μM)^c^	SI^d^
**Azelnidipine**	hT-HFF	VR1814	2.96 ± 0.53	14.65 ± 0.39	192.02 ± 20.46	64.87
	HMVEC		3.09 ± 0.46	18.98 ± 0.27	341.05 ± 20.08	110.37
**Efonidipine**	hT-HFF	VR1814	3.04 ± 0.23	13.58 ± 2.46	339.71 ± 20.10	111.74
	HMVEC		3.59 ± 0.67	19.49 ± 0.72	295.41 ± 11.01	82.28
**Lercanidipine**	hT-HFF	VR1814	3.12 ± 0.43	12.74 ± 1.66	126.54 ± 18.76	40.55
	HMVEC		2.70 ± 0.54	14.53 ± 0.19	285.83 ± 19.06	105.86
**Niguldipine**	hT-HFF	VR1814	3.16 ± 0.64	15.00 ± 0.48	241.16 ± 23.89	76.31
	HMVEC		3.02 ± 0.16	12.74 ± 0.16	205.16 ± 23.30	67.93

a EC_50_, compound concentration that inhibits 50% of VR1814 strain replication, as measured by FFRA in hT-HFFs or HMVECs by FFRAs.

b EC_90_, compound concentration that inhibits 90% of VR1814 strain replication, as determined by FFRA in hT-HFFs or HMVECs by FFRAs.

c CC_50_, compound concentration that produces 50% of cytotoxicity, as determined by cell viability assays in hT-HFFs and HMVEC cells. Reported values represent the means ± SD of data derived from three experiments in duplicate.

d SI, selectivity index determined as the ratio between CC_50_ and EC_50_.

### 3.4 Inhibition of Ca^2+^ channel function of pUS21 by selected CCBs

**Fig. 7:**
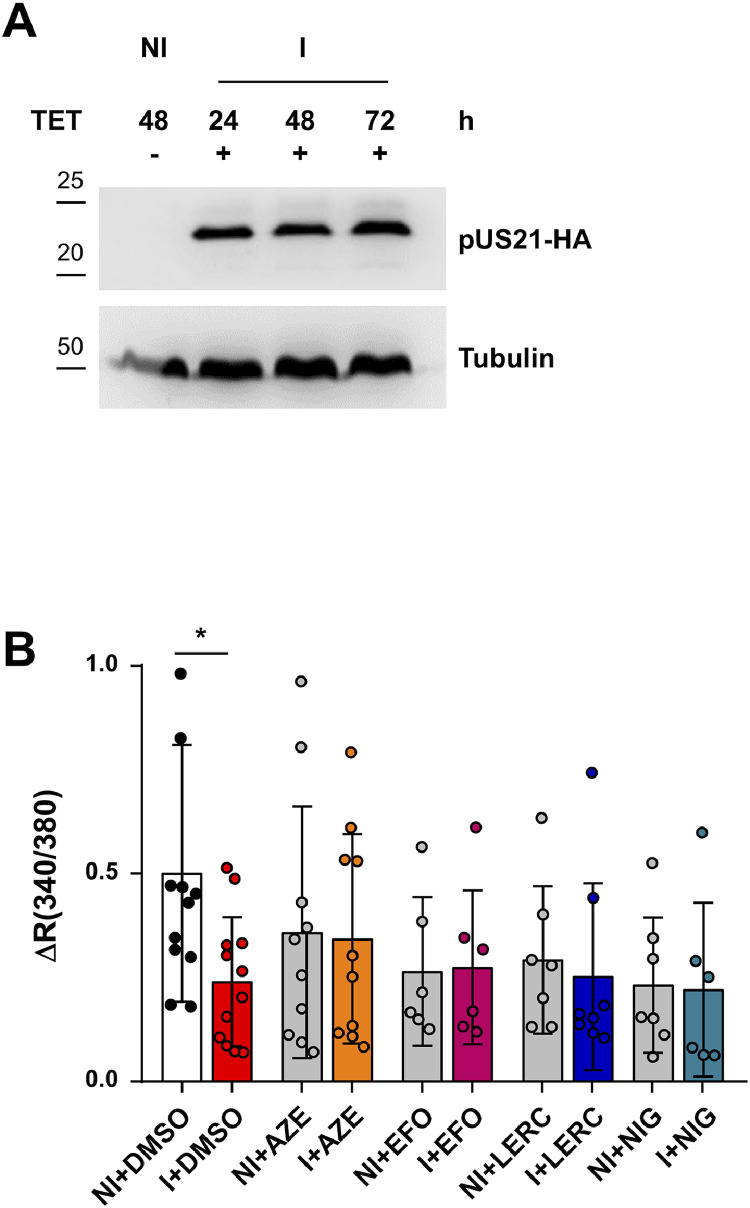
**The selected CCBs abrogate the Ca^2+^-conducting activity of the US21 protein. (A)** Tetracycline-inducible expression of pUS21-HA in T-REx-U2OS-US21-HA cells as detected immunoblotting with an anti-HA mAb. Total cell protein extracts were from cells that were non-induced (NI) or induced (I) with tetracycline 1 μg/ml for 24, 48 and 72 h. Tubulin immunodetection was used as a control for protein loading. **(B)** T-REx-U2OS-US21-HA cells were left uninduced (NI) or induced with tetracycline 1 μg/ml for 48 h and then treated with DMSO as a control, or with 2 x EC_50_ (Table 3) of azelnidipine (AZE), efonidipine (EFO), lercanidipine (LERC), or with niguldipine (NIG). Histograms represent the means ± SD of peak release of Ca^2+^ from the ER following stimulation of cells with IP3mix. Data are from three biological independent experiments, each performed in two technical replicates and analyzed by unpaired *t*-test: *p < 0.05.

*In silico* analysis suggested that the four selected CCBs are capable of establishing different types of interactions with amino acid residues located in the pore of the US21 protein (Fig. 1). Since CCBs block Ca^2+^ channels, we hypothesized that the four selected CCBs might interfere with the Ca^2+^ channel function of pUS21. To experimentally verify this hypothesis, we investigated the effects of CCB treatment on the alteration of intracellular Ca^2+^ homeostasis caused by the ectopic expression of pUS21, which reduces the Ca^2+^content of ER stores (Luganini et al., 2018, 2023). To this end, we performed Ca^2+^ imaging experiments using the ratiometric cytoplasmic Ca^2+^ indicator FURA-2 on cell acutely perfused with the four CCBs. These assays were carried out in T-REx-U2OS-US21-HA cells, which express a HA-tagged pUS21 protein in a tetracycline-inducible manner (Luganini et al., 2023) (Fig. 7A), therefore avoiding any adverse effects on host cell physiology that may be derived from the prolonged constitutive expression of the viroporin. The amount of Ca^2+^ in intracellular ER stores was evaluated indirectly using a Ca^2+^ add-back protocol that measures Ca^2+^ release from the ER following activation of IP3 receptors and inhibition of SERCA ATPase pumps (IP3mix treatment) in a Ca^2+^ free extracellular medium. Ca^2+^-imaging traces (Fig. S3A) and peak amplitude quantification (Fig. 7B) showed that, as previously observed (Luganini et al., 2023), TET-induced expression of pUS21 (I) significantly decreased the amount of Ca^2+^released from the ER stores compared to non-induced (NI) T-REx-U2OS-US21-HA cells, confirming the function of pUS21 as a Ca^2+^ permeable leakage channel (Fig. 7B, Fig. S3A). Notably, the Ca^2+^conducting activity of pUS21, which leads to passive Ca^2+^ leakage from the ER, was abolished by acute perfusion with various CCBs. In fact, following CCB treatment, Ca^2+^ release from the ER was comparable to that of non-induced T-REx-U2OS-US21-HA cells (Fig. 7B, Fig. S3B-E). Importantly, acute perfusion with CCBs did not affect basal Ca^2+^ levels (Fig. S3F), excluding a nonspecific effect of these CCBs on basal Ca^2+^ homeostasis.

Taken together, these results suggest that AZE, EFO, LERC, and NIG can hamper the Ca^2+^-mobilizing activity of pUS21.

### 3.5 CCBs prevent the cytobiological consequences of pUS21 expression

Previously, we observed that the expression of pUS21, by altering intracellular Ca^2+^ homeostasis, increased the resistance of cells to intrinsic apoptosis stimuli and induced cell migration (Luganini et al., 2018, 2023). Given that the four antiviral CCBs hindered pUS21 viroporin activity (Fig. 7), we investigated whether these compounds interfered with the aforementioned cytobiological effects that follow pUS21 expression.

**Fig. 8:**
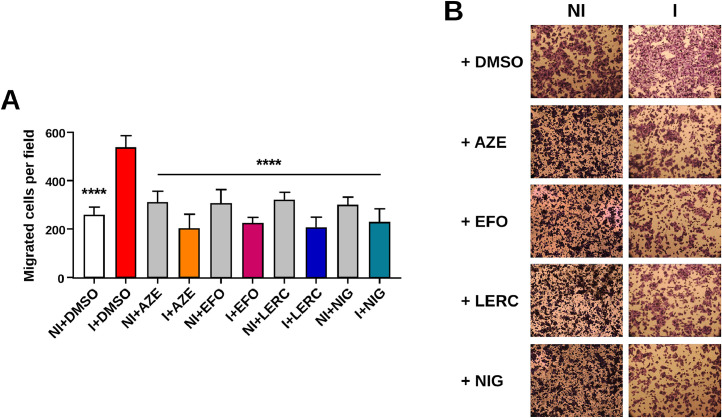
**CCBs hinder the US21-induced cell migration**. T-REx-U2OS-US21-HA cells were left non-induced (NI) or induced (I) with tetracycline (1 μg/ml) for 48 h. Then cells were seeded in the upper chamber of a transwell and incubated at 37 °C in DMEM with the addition, where indicated, of 2 x EC_50_ (Table 3) of AZE, EFO, LERC or NIG, or DMSO, as a control. The lower chamber was filled with DMEM + 10% FBS, and the indicated CCBs were added as described above. After 7 h, cells were fixed and stained with 1% crystal violet. **(A)** Quantification of T-REx-U2OS-US21-HA cell migration as analyzed in at least ten random cell fields. Data shown as means ± SD are from three biological independent experiments, each performed in two technical replicates and analyzed by the Kruskal-Wallis multiple comparison test. **** p < 0.0001 vs calibrator sample (DMSO-treated induced cells). **(B)** Representative images of migrating T-REx-U2OS-US21-HA cells stained with 1% crystal violet. Magnification, × 10.

Regarding the pUS21’s ability to stimulate cell migration, T-REx-U2OS-US21-HA cells were either left non-induced (NI) or induced (I) with tetracycline for 48 h and then treated with the different CCBs at concentrations equal to 2 x EC_50_ (Table 3) before assessing directional cell migration through transwell. As shown in Fig. 8, the inducible expression of pUS21 stimulated the migration of T-REx-U2OS-US21-HA cells, as expected (Luganini et al., 2023). However, treatment of cells expressing pUS21 with the four CCBs prevented viroporin-mediated stimulation of cell migration (Fig. 8). The effect of CCBs is consistent with an interference with pUS21 function, since off-target activity or effects on cellular Ca^2+^ channels would have caused a significant reduction in cell migration even in cells treated with the compounds but not induced to express pUS21. Notably, a similar inhibitory effect of CCBs on pUS21-mediated cell migration was observed in hT-HFFs infected with TRwt (Fig. S4). The lack of stimulation of hT-HFFs migration following infection with a US21-deficient virus (TRUS21) supports the role of viroporin in bestowing the cell response (Fig. S4).

**Fig. 9:**
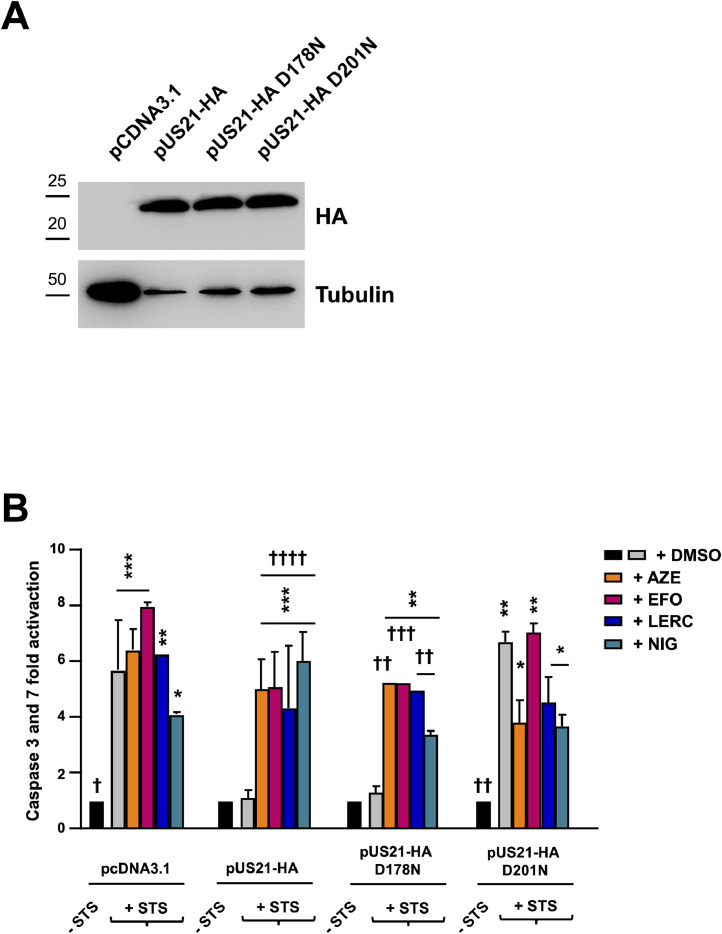
Effect of CCBs on pUS21-mediated protection from intrinsic apoptosis. **(A)** U2OS cells were transfected with empty pcDNA3.1, pUS21-HA, pUS21-HA D178N, or pUS21-HA D201N. At 48 h post-transfection, the expression of pUS21 proteins was confirmed by immunoblotting with an anti-HA mAb. Tubulin immunodetection was used as a control for protein loading. **(B)** Transfected cells were either mock-treated (- STS) or treated with staurosporine (STS) (+ STS) (3 μM, 4 h, 37 °C) in the presence of 2 x EC_50_ CCBs (Table 3) before measuring caspase-3 and caspase-7 activation. Data shown are the means of three biological independent experiments, each performed in two technical replicates ± SD and analyzed by the Dunnett’s multiple comparison test. * p < 0.05; ** p < 0.01; *** p < 0.001; **** p < 0.0001 vs calibrator sample (not STS-treated cells). † p < 0.05; †† p < 0.01; ††† p < 0.001; †††† p < 0.0001 vs calibrator sample (STS-treated cells).

Moreover, because we previously observed that the pUS21-mediated reduction of ER Ca^2+^ content correlated with a protective effect against pro-apoptotic signals (Luganini et al., 2018), we examined whether CCBs also affected this consequence of viroporin activity. To this end, U2OS cells were transiently transfected with pUS21-HA or its single-point mutants US21-HA D178N and US21-HA D201N. Asp178 and Asp201 are located within the pUS21 pore and constitute the TMBIM family conserved pH sensor of pUS21. In particular, while D178N mutation does not affect viroporin activity, D201N is essential since its mutation abolishes the ability of pUS21 to reduce the Ca^2+^content of ER, protect cells from apoptosis and stimulate cell migration and adhesion (Luganini et al., 2018, 2023). At 48 h post-transfection, U2OS cells were exposed to staurosporine (STS) to activate the intrinsic apoptotic pathway, which was subsequently measured by assessing the levels of caspase-3 and caspase-7. As shown in Fig. 9, the transient expression of pUS21-HA and pUS21-HA D218N significantly reduced the STS-induced caspase 3 and 7 activation compared with the empty pcDNA3.1. The expression of the non-functional pUS21-HA D201N protein did not decrease the ability of pUS21 to protect cells from intrinsic apoptosis, as expected (Luganini et al., 2018). However, the addition of 2 x EC_50_ CCBs reversed the pUS21-mediated protective effect of pUS21-HA and pUS21-HA D218N, restoring the activation of caspase 3 and 7, indicating that their interference with the Ca^2+^-conducting activity of pUS21 abolished the viroporin protection effect.

These results further support the involvement of pUS21 in the action of selected CCBs against the US21 viroporin function, resulting in the inhibition of the relevant cytobiological consequences of pUS21 function that contribute to efficient HCMV replication and pathogenesis.

### 3.6 CCBs and maribavir operate synergistically against the replication of GCV-resistant HCMV

Because currently approved antiviral drugs for the treatment of HCMV infections have significant limitations, we evaluated the effect of a drug combination between each selected dihydropyridine CCBs and maribavir (MBV), an inhibitor of the viral UL97 kinase approved in 2021 for clinical use. Nevertheless, MBV exhibits a clinically relevant side effect as it increases the plasma concentration of immunosuppressants usually used in transplant recipients (Burger et al., 2024), which, in turn, upregulates blood pressure, a major risk factor for organ rejection (Hoorn et al., 2011, 2012). Therefore, anti-hypertensive CCBs, such as AZE, EFO, LERC and NIG, may provide a dual therapeutic effect by exerting anti-HCMV activity and mitigating MBV-associated hypertensive risk in transplant recipient endager by HCMV reactivation. To investigate the effects of this drug combinations in the relevant cell model of endothelial cells, focus-forming reduction assays (FFRAs) were performed with the GCV-resistant HCMV TRwt strain (Smith et al., 1998) in HMVECs treated with different concentrations of both each CCB and MBV corresponding to 0.25-, 0.5-, 1-, 2-, or 4-fold of their EC_50_ values to achieve equipotent ratios (CCB EC_50_/MBV EC_50_). As reported in Fig. S5, S6 and Table 5, a synergistic effect for the combination of MBV with each of all four drugs tested was observed, since the Combination Index (CI) resulted <0.7 (Chou, 2006), even when the two drugs were combined at 0.25-fold of their respective EC_50s_ (Table 5). The synergic effect increased progressively, reaching a powerful level when the combination was equal to 4-fold the respective EC_50_ values. Importantly, none of the CCB-MBV combinations exerted a significant cytotoxic activity against HMVECs (Table 5), indicating that their synergistic anti-HCMV activity was not the result of an increased cytotoxicity, but that it derived from the combined interference with different targets and mechanisms of action.

**Table 5:** Analysis of the effects of the combination of each CCB and maribavir on HCMV TRwt virus replication and cell viability.

AZE + MBV Combination at equipotent ratio (fold of EC_50_)^a^	Fa^b^	AZE + MBV CI^c^	Drug combination Effect^d^ AZE + MBV	% of Cell Viability
4x	0.31 ± 0.04	4.33×10^–8^± 0.01	Very Strong Synergism	84.14 ± 1.43
2x	0.47 ± 0.03	0.23 ± 0.01	Strong Synergism	89.61 ± 1.86
1x	0.61 ± 0.13	0.32 ± 0.02	Synergism	92.82 ± 2.25
0.5x	0.69 ± 0.07	0.36 ± 0.18	Synergism	95.91 ± 3.93
0.25x	0.75 ± 0.04	0.58 ± 0.04	Synergism	99.01 ± 0.08

EFO + MBV Combination at equipotent ratio (fold of EC_50_)^a^	Fa^b^	EFO + MBV CI^c^	Drug combination Effect^d^ EFO + MBV	% of Cell Viability
4x	0.29 ± 0.03	8.68×10^–7^± 0.01	Very Strong Synergism	81.68 ± 1.22
2x	0.53 ± 0.06	0.004 ± 0.02	Very Strong Synergism	88.20 ± 1.59
1x	0.66 ± 0.01	0.220 ± 0.03	Strong Synergism	93.76 ± 0.47
0.5x	0.93 ± 0.01	0.510 ± 0.21	Synergism	95.11 ± 0.46
0.25x	0.95 ± 0.06	0.410 ± 0.01	Synergism	94.01 ± 4.81

LERC + MBV Combination at equipotent ratio (fold of EC_50_)^a^	Fa^b^	LERC + MBV CI^c^	Drug combination Effect^d^ LERC + MBV	% of Cell Viability
4x	0.29 ± 0.03	6.33×10^–8^± 0.03	Very Strong Synergism	83.22 ± 2.57
2x	0.43 ± 0.03	0.35 ± 0.10	Synergism	92.99 ± 5.66
1x	0.57 ± 0.81	0.35 ± 0.10	Synergism	91.88 ± 4.16
0.5x	0.69 ± 0.07	0.30 ± 0.02	Synergism	93.91 ± 4.99
0.25x	0.85 ± 0.07	0.44 ± 0.04	Synergism	97.19 ± 0.75

NIG + MBV Combination at equipotent ratio (fold of EC_50_)^a^	Fa^b^	NIG + MBV Cic	Drug combination Effect^d^ NIG + MBV	% of Cell Viability
4x	0.09 ± 0.04	3.13×10^–6^± 0.0001	Very Strong Synergism	82.02 ± 1.73
2x	0.13 ± 0.08	0.47 ± 0.02	Synergism	90.05 ± 1.70
1x	0.46 ± 0.06	0.47 ± 0.04	Synergism	94.12 ± 3.12
0.5x	0.73 ± 0.06	0.43 ± 0.04	Synergism	97.18 ± 0.78
0.25x	0.85 ± 0.03	0.30 ± 0.03	Synergism	99.22 ± 2.10

a Fold increase of EC_50_ of each CCBs/EC_50_ maribavir (MBV) yielding an equipotent ratio concentration between the two combined drugs. The EC_50s_ for each CCBs were determined by FFRA against TRwt in HMVEC cells, as described in Material and Methods. EC_50_ values were: for AZE, 2.12 μM; for EFO, 3.55 μM; for LERC, 3.32 μM; for NIG, 1.15 μM (Table 3); for MBV 4.74 μM (data not shown). The ratio concentrations were considered for AZE, 1:0.44; for EFO, 1:0.74; for LERC, 1:0.70; for NIG, 1:0.24.

b Fa, fractional effect analysis.

c Combination Index (CI), obtained by computational analysis with Calcusyn software and based on dose-effect data. Reported values are showed as the means ± SD of data derived from two independent biological experiments, each conducted in three technical replicates using CCB+MBV concentration ratio as showed in ^a^.

d Drug combination effect defined as: very strong synergism for < 0.1; strong synergism for 0.1<CI<0.3; synergism for 0.3<CI<0.7 according to Chou (2010).

## 4 Discussion

The functional characterization of regulatory viral proteins, such as the US21 viroporin of HCMV, not only supports a better understanding of viral replication and pathogenesis, but may also open novel avenues for the identification and development of antiviral strategies that exploit these functions.

The present study validated this hypothesis by identifying four dihydropyridine Ca^2+^ antagonists that inhibit pUS21 Ca^2+^-conducting activity and hamper HCMV replication in various cell lines. These CCB compounds (azelnidipine, efonidipine, lercanidipine, and niguldipine) have been widely characterized for their pharmacological profiles and, with the exception of niguldipine, have been approved for clinical use as antihypertensive drugs (Frishman, 2007; McKeever et al., 2024). Azelnidipine blocks l-type Ca^2+^ channels and has been approved by the Pharmaceuticals and Medical Devices Agency (PMDA) in Japan since 2003 for clinical use in arterial hypertension (Wellington and Scott, 2003). Efonidipine inhibits both l- and T-type Ca^2+^ channels, thereby exerting a dual mechanism that affects both blood pressure and heart rate. It has been approved by the PMDA in Japan in the mid-1990s for the treatment of arterial hypertension and, in some Asian clinical settings, also for angina (Shimizu et al., 2003). Lercanidipine is another l-type Ca²⁺ channel blocker that was approved in 1997 by the European Medicines Agency (EMA) for the treatment of hypertension (Epstein, 2001). Niguldipine preferentially blocks l-type channels and, at certain concentrations, also T-type Ca²⁺ channels. However, it has not yet been approved for clinical use in humans (Boer et al., 1989). Notably, our results indicate that these CCBs can be repositioned as antiviral agents that target HCMV through a mechanism of action different from those of the currently available anti-HCMV drugs (Britt and Prichard, 2018; Piret and Boivin, 2019; Gourin et al., 2023).

The anti-HCMV CCBs were identified using an *in silico* structure-based virtual screening that exploited the predicted structure of pUS21 (Fig.1A). Docking simulations revealed energetically favorable binding poses for the four CCBs, and their direct interactions with specific amino acid residues located within the inner region of the pUS21 pore were predicted by bioinformatic analysis (Fig. 1B-E). These interactions were further supported by MD simulations, which confirmed the stability of the ligand-protein complexes over time (Fig. 2, Fig. 3). Although MD simulations do not provide direct evidence of binding affinity or specificity, they provide insights into the dynamic behavior of ligand-protein complexes. In our case, the selected CCBs maintained stable conformations within the pUS21 pore, whereas the weak CCB binder, felodipine, showed higher mobility and conformational drift. Thus, MD simulations should be considered complementary rather than definitive evidence of binding and specificity.

Given that pUS21 is required for efficient HCMV replication in different cell types (Luganini et al., 2018), it is not unexpected that all four selected compounds showed dose-dependent antiviral activity, with EC_50_ in the low micromolar range, which was neither cell type- nor virus strain-specific (Table 3, Table 4). Notably, the observation that the EC_50_ values measured with a US21-deficient virus increased by more than two-fold compared to those of TRwt (Table 3) supports the view that the overall anti-HCMV activity of the selected CCBs may be derived, at least in part, from the specific targeting of pUS21 and the interference with its viroporin activity, perhaps by adding or synergizing with the inhibitory effects of the CCBs on cellular Ca^2+^ channels. Given that the Ca^2+^ channel activity of pUS21 is required for the pUS21-mediated increase in cell resistance to intrinsic apoptosis (Luganini et al., 2018), the interference of selected CCBs with the pUS21-dependent anti-apoptotic effect (Fig. 9) may hamper the survival of infected cells, thereby shortening the viral replication and reducing HCMV yield.

The finding that CCBs restored the normal ER Ca^2+^content in cells overexpressing pUS21 provided further functional evidence of their specificity for the HCMV viroporin (Fig. 7).

However, it is worth discussing the apparent discrepancy between the low micromolar CCB EC_50_ values measured *in vitro* (Table 3, Table 4) and their nanomolar plasma concentrations that can be achieved following therapeutic dosing (Liu et al., 2015; Chaudhary et al., 2016; Chen et al., 2024). Dihydropyridine CCBs are highly lipophilic compounds with the potential to accumulate in lipid membranes (Seydel et al., 1994) and are characterized by high plasma protein binding, large volumes of distribution, and wide tissue diffusion, rather than remaining solely in the plasma compartment (Kelly and O'Malley, 1992; Sesin and Tamargo, 1997; Godfraind, 2017). Therefore, these pharmacokinetic properties suggest that CCBs may accumulate in cellular membranes at concentrations higher than in plasma, potentially reaching or locally exceeding the EC_50_ values measured *in vitro* (Table 3, Table 4). However, because direct *in vivo* pharmacokinetic evidence for such exposure levels is currently lacking, the CCBs analyzed here may be considered leads rather than immediate therapeutic candidates. Further optimization, including structure-guided medicinal chemistry to improve potency and pharmacokinetic properties, is required to increase their translational feasibility beyond the *in vitro* experimental setting.

Recently, some approved CCBs have been observed to exert antiviral effects against human viruses. Indeed, verapamil has been reported to block Ebola virus entry by acting on two-pore channels (Sakurai et al., 2015), whereas Wang et al. (2022) observed that diltiazem reduces SARS-CoV-2 infection by interfering with the l-type Ca^2+^-channel Cav1.2, which is involved in viral entry. Similarly, Li et al. (2025) reported that the CCB cilnidipine inhibits Influenza A virus entry and fusion both *in vitro* and *in vivo* by interfering with Cav1.2 and Cav2.1 function.

However, unlike these previous observations, the results of this study provide the first evidence consistent with the possibility that CCBs may interfere with a viral-encoded Ca^2+^ channel, opening new avenues for the development of specific CCB-based viroporin-targeted antiviral strategies. Thus, our study contributes to the rapidly developing field of investigation that exploits viroporins as novel and preferential virus-specific drug targets, whose importance is supported by their essential roles in tuning efficient viral replication and contributing to virus pathogenesis (Nieva et al., 2012; Breitinger et al., 2022; Devantier et al., 2024; Alcaraz and Nieva, 2025; Qu et al., 2025; Peng et al., 2025).

Furthermore, our study adds a new piece of knowledge that may be relevant to the development of dihydropyridine Ca^2+^-antagonists against HCMV infections and pertains to the observation that a combination of CCBs and maribavir (MBV), a recently approved inhibitor of HCMV UL97 kinase, is synergistic against HCMV replication (Table 5). This combination was investigated because of the reported increase in the plasma concentration of immunosuppressants in transplant recipients administered MBV (Burger et al., 2024), which increases blood pressure and may be a major risk factor for organ rejection (Hoorn et al., 2011, 2012). This MBV side effect is particularly relevant in transplant recipients or patients undergoing chronic immunosuppressive therapy, who represent the main population at risk of HCMV reactivation and require anti-HCMV therapy. Therefore, the observation of the efficacy of the combination of MBV and CCBs could suggest the design of therapeutic regimens with reduced MBV doses, which not only reduces the risk of selection of viral resistance but also limits the increase in blood pressure (Hoorn et al., 2011, 2012; Burger et al., 2024). Moreover, the management of HCMV infections in hypertensive patients may benefit from dihydropyridine CCB treatment not only to achieve better control of blood pressure and improve endothelial cell function, but also to reduce vascular inflammation, which may be exacerbated by HCMV infection (Cheng et al., 2009). Overall, the combination of their cardiovascular and antiviral properties further strengthens the potential of the selected dihydropyridine CCBs as complementary tools for integrated anti-HCMV strategies in transplant recipients.

## 5 Conclusions

In conclusion, this study validates pUS21 as a novel HCMV-specific target for antiviral drug discovery, as the inhibition of its viroporin activity represents an effective strategy to control the proviral cytobiological consequences of pUS21-mediated alterations in Ca^2+^ homeostasis in HCMV-infected cells. Notably, CCBs repositioned as anti-HCMV agents show advantageous features, such as potent antiviral activity even against virus strains resistant to conventional polymerase inhibitors, amenability to combination regimens with approved antiviral drugs with new targets, such as MBV, and the availability of already accomplished clinical development processes that may reduce cost and timeline to clinical evaluation as antivirals, even in settings where pharmacological treatment of HCMV infections is currently unavailable.

## CRediT authorship contribution statement

**Giulia Sibille:** Methodology, Investigation, Validation, Formal analysis, Visualization, Writing-review & editing. **Davide Loggia:** Investigation, Formal analysis, Visualization, Writing-review & editing. **Gianluca Catucci:** Methodology, Investigation, Formal analysis, Visualization. **Alessandra Gilardino:** Methodology, Validation, Formal analysis**. Alessandra Fiorio Pla:** Investigation, Formal analysis, Visualization. **Giovanna Di Nardo:** Investigation, Formal analysis, Visualization. **Luca Munaron:** Resources, Formal analysis. **Gianfranco Gilardi:** Resources, Formal analysis. **Giorgio Gribaudo:** Conceptualization, Validation, Visualization, Writing-original draft, Writing-review & editing, Funding acquisition. **Anna Luganini:** Conceptualization, Validation, Visualization, Writing-original draft, Writing-review & editing, Supervision, Project administration, Funding acquisition.

## Funding

This work was supported by the European Union - Next Generation EU (POC PNRR TOINPROVE/2023) to A.L.; a research grant from the Foundation “Istituto di Ricerca Virologica Oretta Bartolomei Corsi”, Florence (Italy) to A.L.; and the EU funding within the MUR PNRR Extended Partnership Initiative on Emerging Infectious Diseases (Project No PE00000007, INF-ACT) to G. Gribaudo.

## Declaration of competing interest

The authors declare the following financial interests/personal relationships which may be considered as potential competing interests: Anna Luganini, Giorgio Gribaudo, Alessandra Fiorio Pla, Luca Munaron, Giovanna Di Nardo, Gianfranco Gilardi has patent #n:102,025,000,001,680 issued to University of Turin. If there are other authors, they declare that they have no known competing financial interests or personal relationships that could have appeared to influence the work reported in this paper.

## Appendix Supplementary materials

**Figure S1. Per-residue pLDDT profile of the AlphaFold-predicted pUS21 structure.** The green, red and blue boxes correspond to pore-lining residues 137–145, 152–178 and 200–211, respectively. The magenta line is set on a score of 60. Image, image 1 dummy alt text

**Figure S2. Predicted Alignment Error (PAE) PAE heatmap of the AlphaFold-predicted pUS21 structure.** The red boxes highlight regions corresponding to pore-lining residues. Image, image 2 dummy alt text

**Figure S3. Cytosolic Ca^2+^ signals upon acute treatment with CCBs. (A-E)** Representative cytosolic Ca^2+^-imaging traces ±SD obtained applying the Ca^2+^ add back protocol. T-REx-U2OS US21-HA cells were non-induced (NI) or induced (I) with 1 μg/ml tetracycline for 48 h, then ER release was induced by activation of IP3 receptors via IP3mix, as described in Material and Methods. Recordings were performed in presence of DMSO as control **(A)**; or after acute treatment with 2 x EC_50_ (Table 3) of azelnidipine **(B);** efonidipine **(C)**; lercanidipine **(D)**; niguldipine **(E). (F)** Basal Ca^2+^ levels quantification (R 340/380) measured in the presence of extracellular Ca^2+^. Data are expressed as mean of Ca^2+^ baseline ± SD and are from three biological independent experiments, each performed in two technical replicates. Image, image 3 dummy alt text

**Figure S4. Effect of CCBs on the pUS21-dependent chemotactic migration of HCMV-infected fibroblasts.** hT-HFFs were subjected to serum starvation for 24 h and then mock-infected or infected with TRwt or TRΔUS21 at an MOI of 1 PFU/cell. Then, cells were subsequently treated with 2 x EC_50_ (Table 3) of AZE, EFO, LERC, NIG or DMSO as a control before being analyzed in chemotactic migration assays, as described in Material and Methods. Data shown are means ± SD of three biological independent experiments, each performed in three technical replicates analyzed by the Dunnett’s multiple comparison test. **** p < 0.0001 vs calibrator sample (DMSO-treated TRwt-infected cells). Image, image 4 dummy alt text

**Figure S5. The combination of CCBs and maribavir is synergistic against the replication of HCMV TRwt in endothelial cells.** FFRAs were perfomed in HMVECs cells infected with the GCV-resistant TRwt strain (50 PFU/well). Cells were exposed to different concentrations of each CCB alone (colored line), maribavir (gray line) or in combination with each other (black dashed line) from −1 h to 96 h p.i. At 96 h p.i., the viral plaques were stained with an anti-IEA mAb and microscopically counted. The effect of the combination was then analyzed by the CompuSyn software and displayed as a fractional effect analysis (Fa) plot in relation to the compound concentrations. (**A**) azelnidipine; (**B**) efonidipine; (**C**) lercanidipine; (**D**) niguldipine. The 2-drug combination studies were performed in two independent biological experiments, each performed in three technical replicates. For the graphical representation, the mean values of CI and Fa were used (Table 5). Image, image 5 dummy alt text

**Figure S6. Combination index-Fa plots and isobologram analysis representing the combination effect of each CCB with maribavir.** The interaction between maribavir (MBV) and the CCBs AZE, EFO, LERC, and NIG was assessed using combination index (CI) analysis and normalized isobolograms. The left panels show CI plots as a function of fractional effect analysis (Fa), where CI values < 1 indicate synergy. The right panels show normalized isobolograms, with each point representing a specific experimentally tested combination of the two drugs. The diagonal line indicates the theoretical additive effect, whereas the experimental points below this line indicate synergistic interactions between the two drugs. Image, image 6 dummy alt text
