# Supplementary figures and images for: Targeting the US21 viroporin of human cytomegalovirus by calcium channel blockers as a new antiviral strategy
Source: Curr Res Microb Sci. 2026 Apr 19;10:100599. doi: 10.1016/j.crmicr.2026.100599 (PMC13129453; doi:10.1016/j.crmicr.2026.100599)

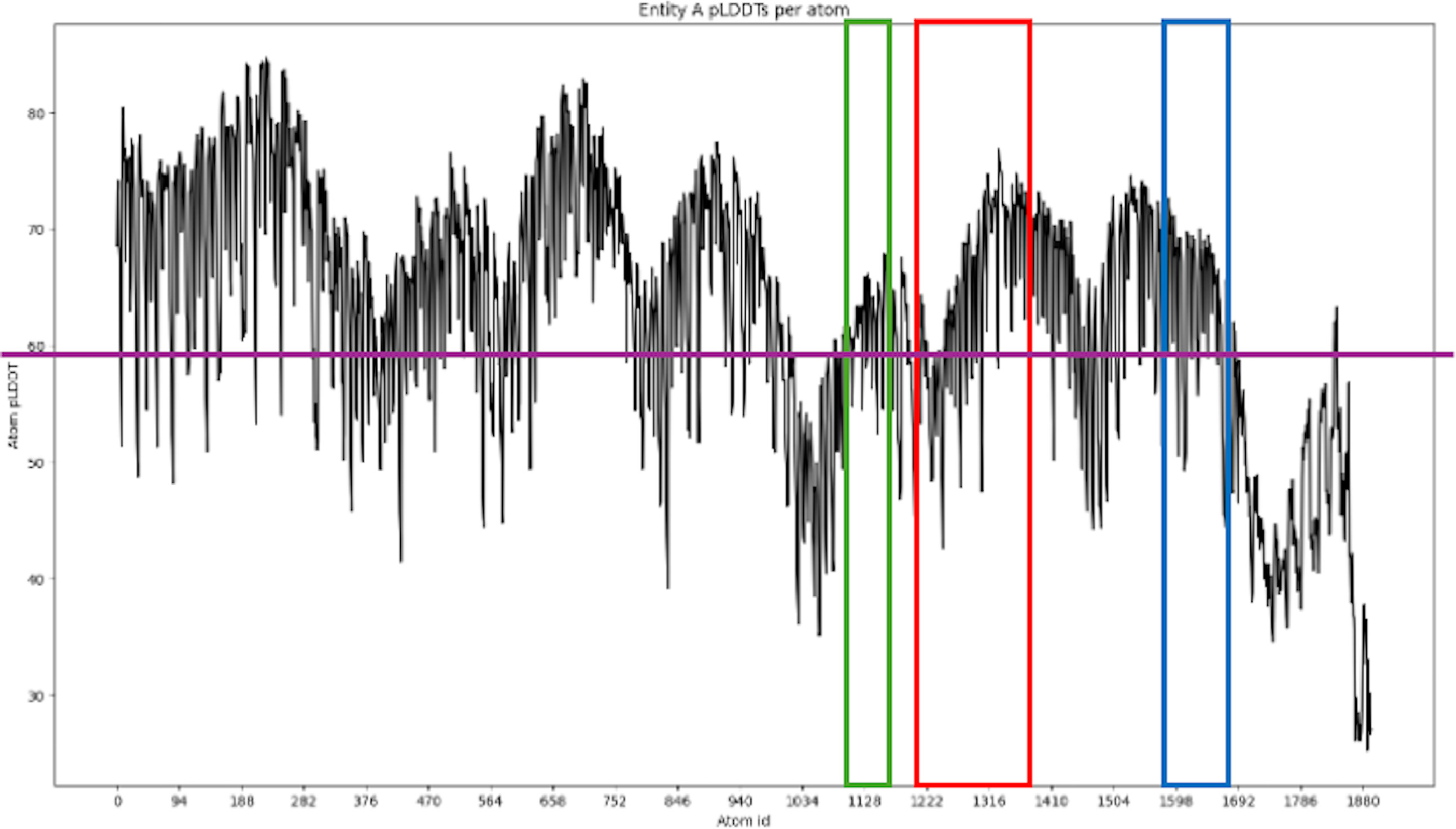

Supplement: Supplementary file 1 — Figure S1. Per-residue pLDDT profile of the AlphaFold-predicted pUS21 structure. The green, red and blue boxes correspond to pore-lining residues 137–145, 152–178 and 200–211, respectively. The magenta line is set on a score of 60. [file mmc1.jpg]

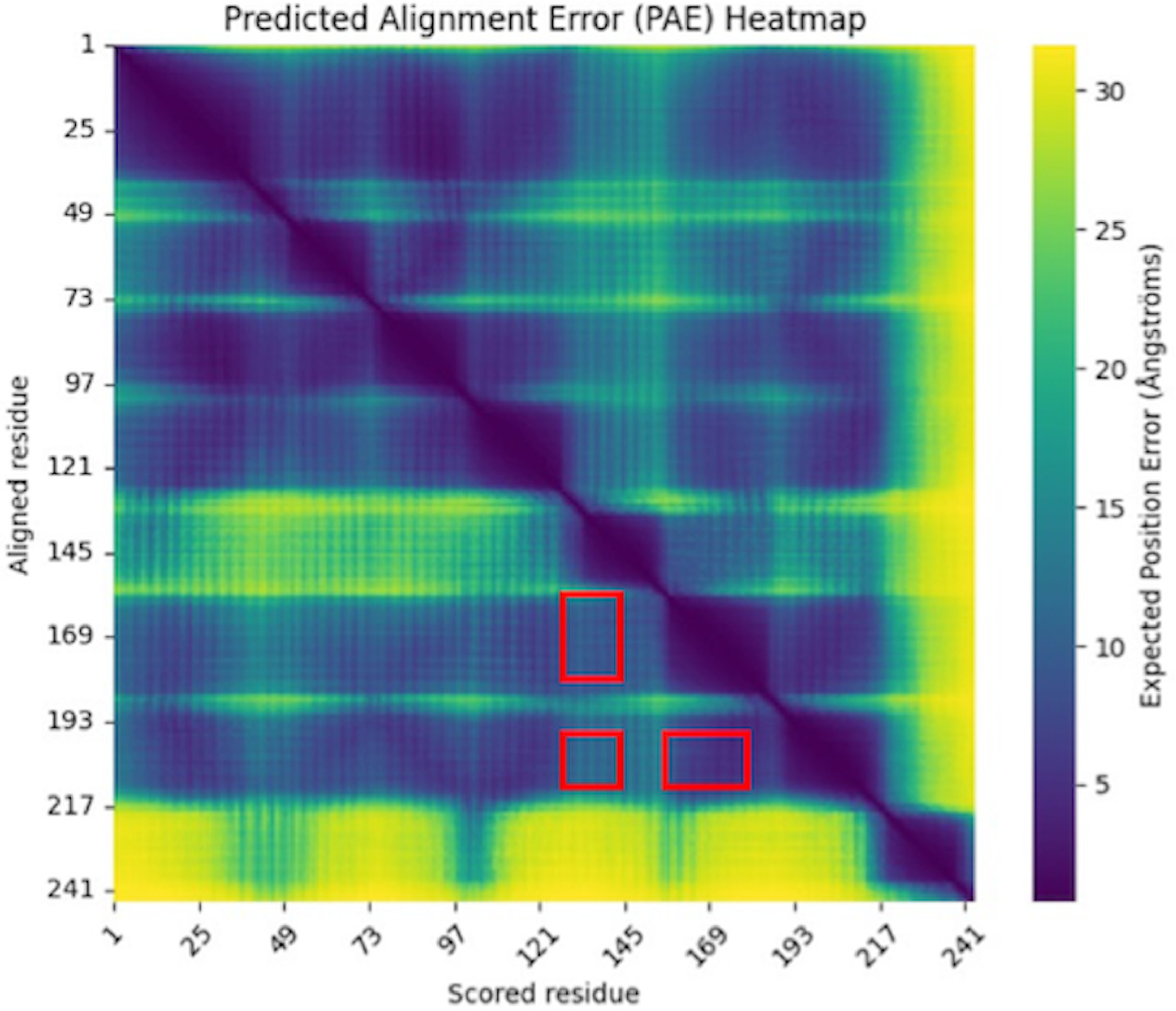

Supplement: Supplementary file 2 — Figure S2. Predicted Alignment Error (PAE) PAE heatmap of the AlphaFold-predicted pUS21 structure. The red boxes highlight regions corresponding to pore-lining residues. [file mmc2.jpg]

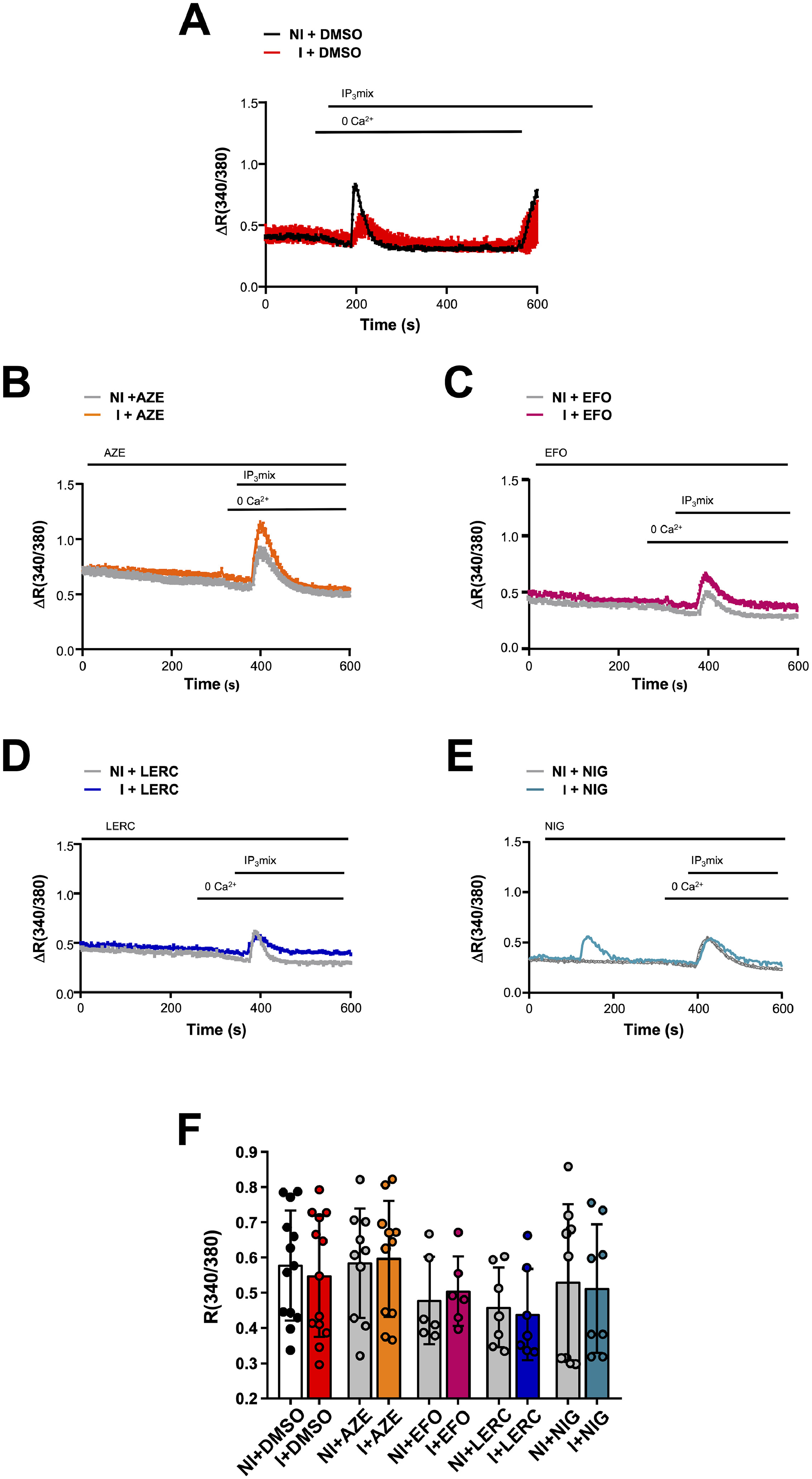

Supplement: Supplementary file 3 — Figure S3. Cytosolic Ca2+ signals upon acute treatment with CCBs. (A-E) Representative cytosolic Ca2+-imaging traces ±SD obtained applying the Ca2+ add back protocol. T-REx-U2OS US21-HA cells were non-induced (NI) or induced (I) with 1 μg/ml tetracycline for 48 h, then ER release was induced by activation of IP3 receptors via IP3mix, as described in Material and Methods. Recordings were performed in presence of DMSO as control (A); or after acute treatment with 2 x EC50 (Table 3) of azelnidipine (B); efonidipine (C); lercanidipine (D); niguldipine (E). (F) Basal Ca2+ levels quantification (R 340/380) measured in the presence of extracellular Ca2+. Data are expressed as mean of Ca2+ baseline ± SD and are from three biological independent experiments, each performed in two technical replicates. [file mmc3.jpg]

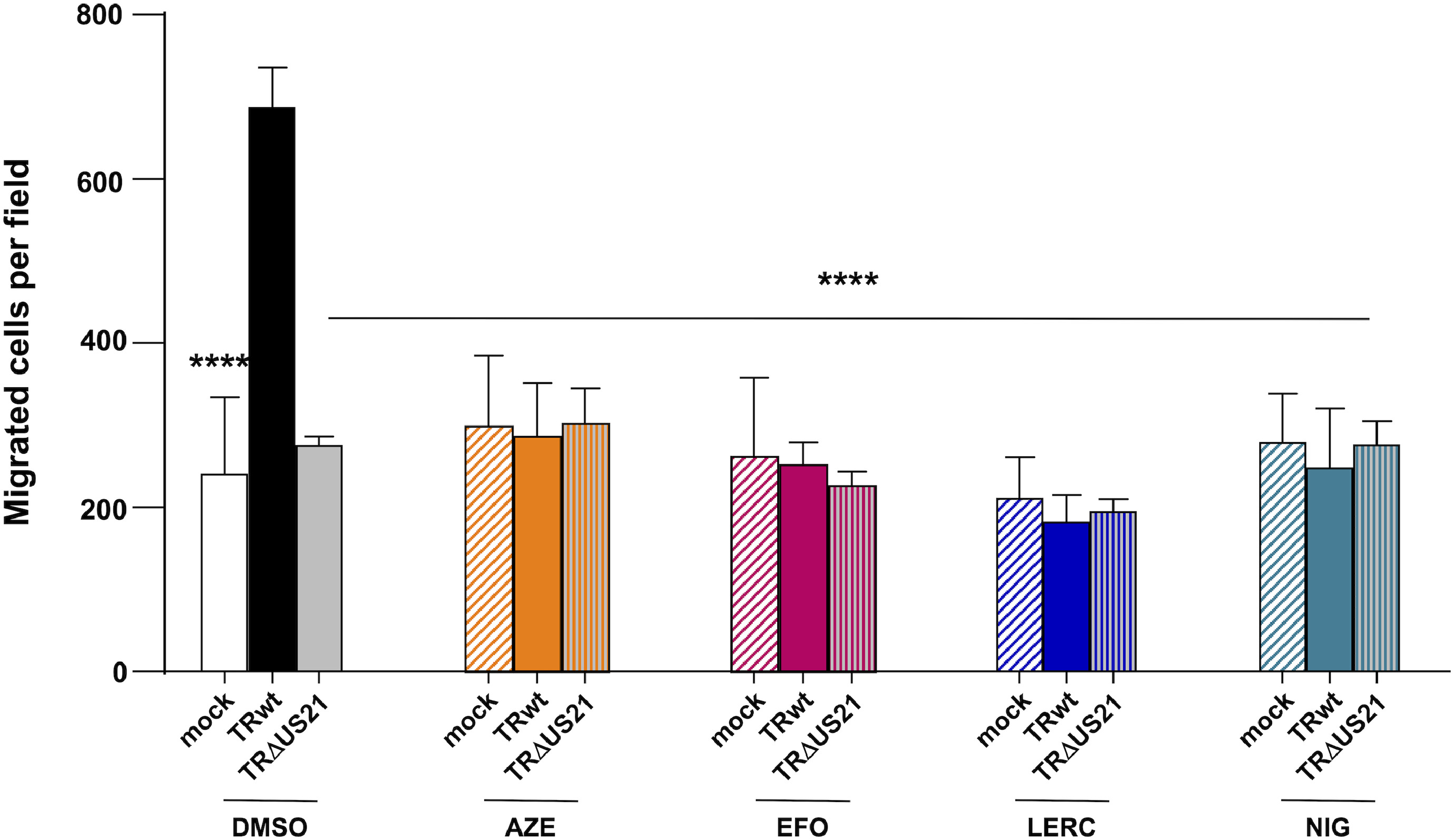

Supplement: Supplementary file 4 — Figure S4. Effect of CCBs on the pUS21-dependent chemotactic migration of HCMV-infected fibroblasts. hT-HFFs were subjected to serum starvation for 24 h and then mock-infected or infected with TRwt or TRΔUS21 at an MOI of 1 PFU/cell. Then, cells were subsequently treated with 2 x EC50 (Table 3) of AZE, EFO, LERC, NIG or DMSO as a control before being analyzed in chemotactic migration assays, as described in Material and Methods. Data shown are means ± SD of three biological independent experiments, each performed in three technical replicates analyzed by the Dunnett’s multiple comparison test. **** p < 0.0001 vs calibrator sample (DMSO-treated TRwt-infected cells). [file mmc4.jpg]

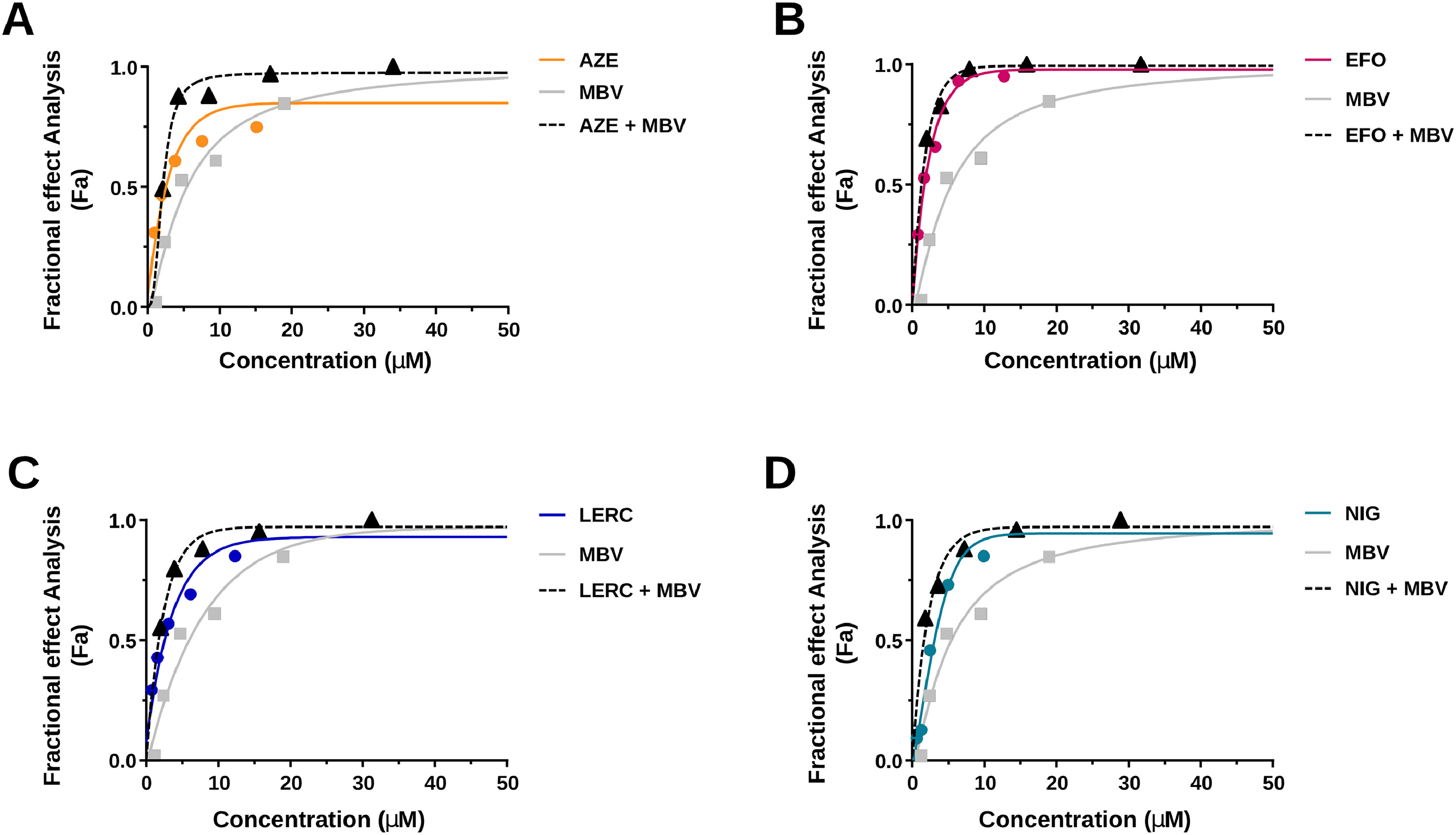

Supplement: Supplementary file 5 — Figure S5. The combination of CCBs and maribavir is synergistic against the replication of HCMV TRwt in endothelial cells. FFRAs were perfomed in HMVECs cells infected with the GCV-resistant TRwt strain (50 PFU/well). Cells were exposed to different concentrations of each CCB alone (colored line), maribavir (gray line) or in combination with each other (black dashed line) from −1 h to 96 h p.i. At 96 h p.i., the viral plaques were stained with an anti-IEA mAb and microscopically counted. The effect of the combination was then analyzed by the CompuSyn software and displayed as a fractional effect analysis (Fa) plot in relation to the compound concentrations. (A) azelnidipine; (B) efonidipine; (C) lercanidipine; (D) niguldipine. The 2-drug combination studies were performed in two independent biological experiments, each performed in three technical replicates. For the graphical representation, the mean values of CI and Fa were used (Table 5). [file mmc5.jpg]

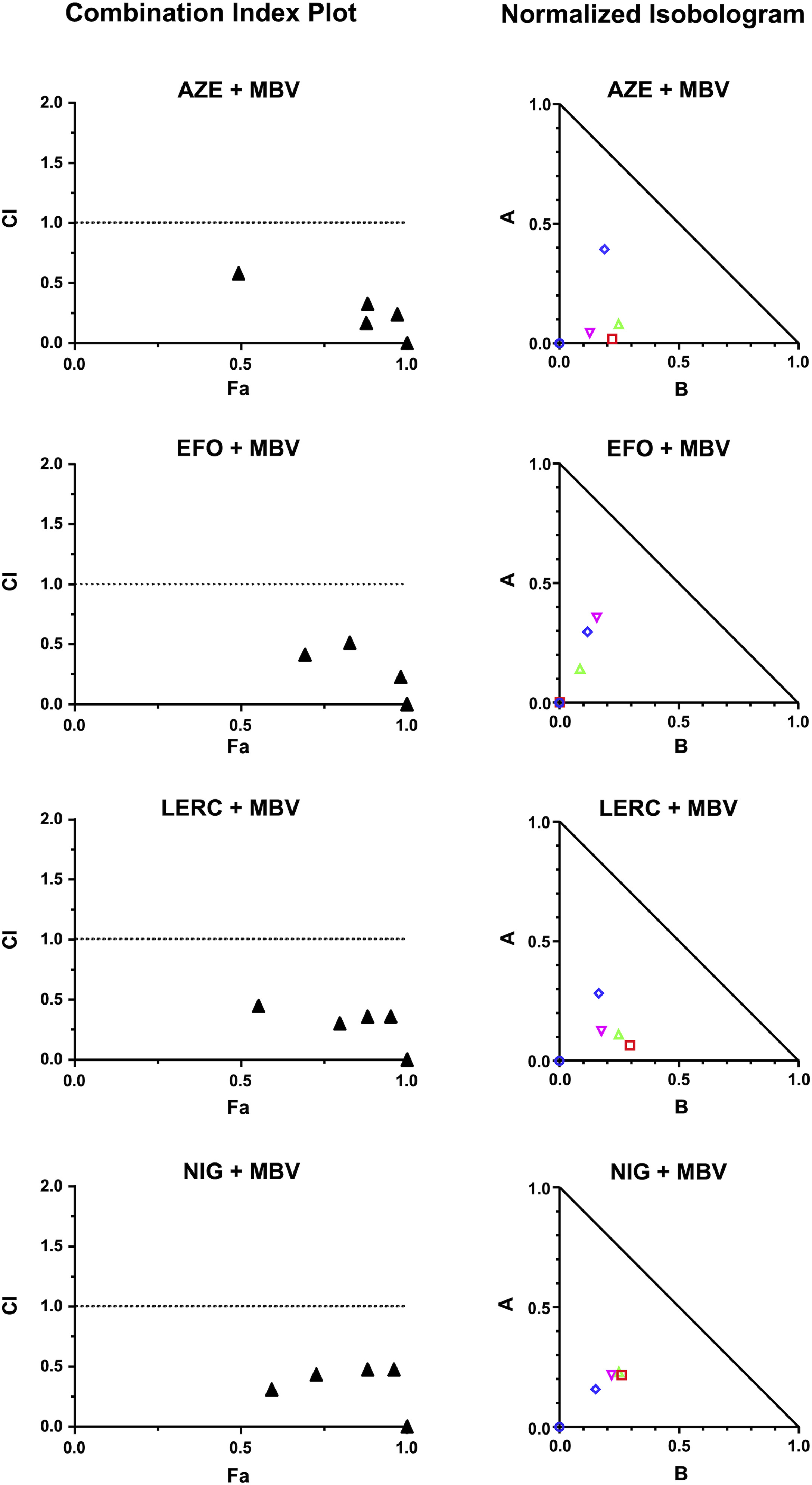

Supplement: Supplementary file 6 — Figure S6. Combination index-Fa plots and isobologram analysis representing the combination effect of each CCB with maribavir. The interaction between maribavir (MBV) and the CCBs AZE, EFO, LERC, and NIG was assessed using combination index (CI) analysis and normalized isobolograms. The left panels show CI plots as a function of fractional effect analysis (Fa), where CI values < 1 indicate synergy. The right panels show normalized isobolograms, with each point representing a specific experimentally tested combination of the two drugs. The diagonal line indicates the theoretical additive effect, whereas the experimental points below this line indicate synergistic interactions between the two drugs. [file mmc6.jpg]
